# Supplementary material for: Next-generation unnatural monosaccharides reveal that ESRRB O-GlcNAcylation regulates pluripotency of mouse embryonic stem cells
Source: Nat Commun. 2019 Sep 6;10:4065. doi: 10.1038/s41467-019-11942-y (PMC6731260; doi:10.1038/s41467-019-11942-y)
Supplement: Supplementary file 1 — Supplementary Information [file 41467_2019_11942_MOESM1_ESM.pdf]

SUPPLEMENTARY INFORMATION

Next-generation unnatural monosaccharides reveal that ESRRB O-GlcNAcylation regulates pluripotency of mouse embryonic stem cells

Hao and Fan, et al

Supplementary Figures

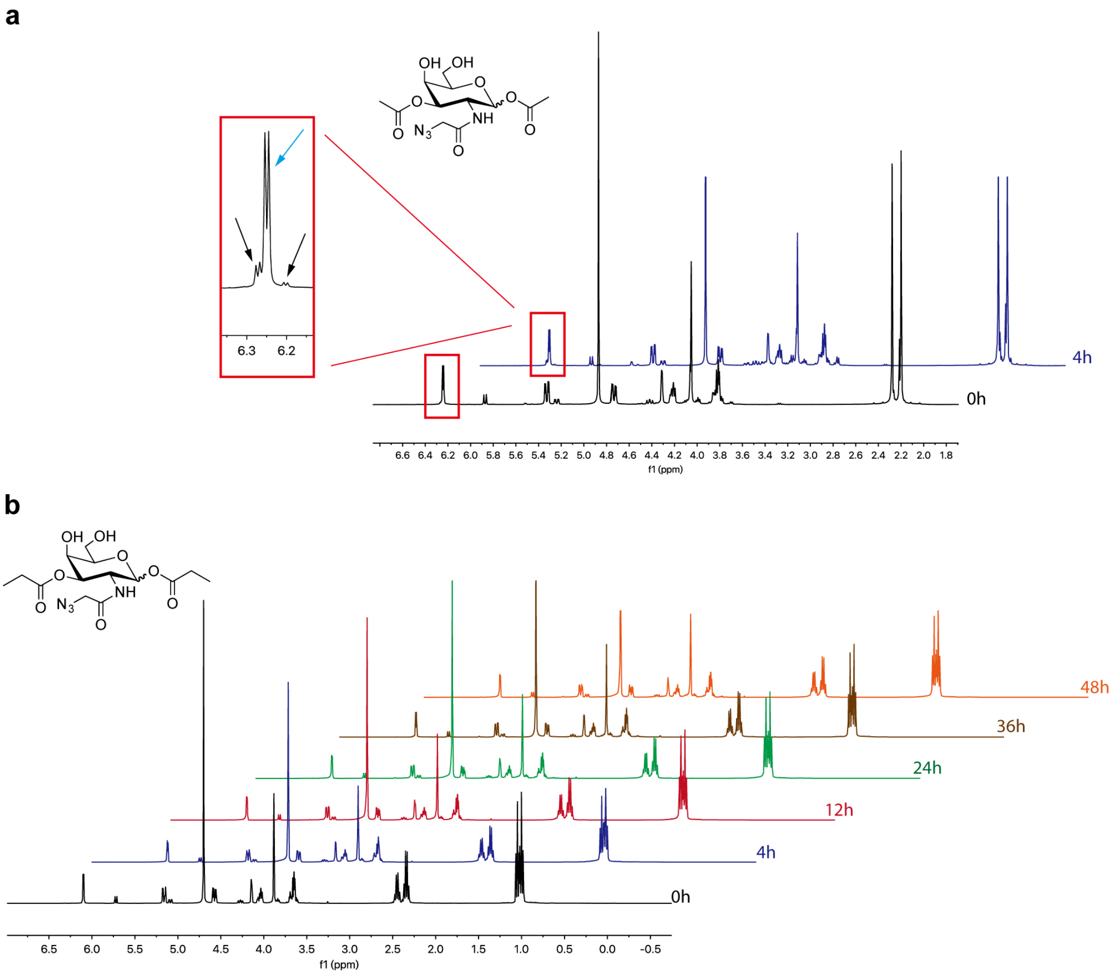

Supplementary Fig. 1 | Migration of acyl groups of **3** and **4**. **a, b,** 1,3-Ac_2GalNAz (**3**) or 1,3-Pr_2GalNAz (**4**) was dissolved in D_2O and monitored by ^{1}H NMR for up to 48 h. The blue arraw indicates the doublet peak of β-H at C-1 of 1,3-Ac_2GalNAz and the black arrows indicate peaks of β-H at C-1 of 1,4-Ac_2GalNAz and 1,6-Ac_2GalNAz, in which the acetyl groups migrated onto the 4- and 6-hydroxyl groups from the 3-hydroxyl position.

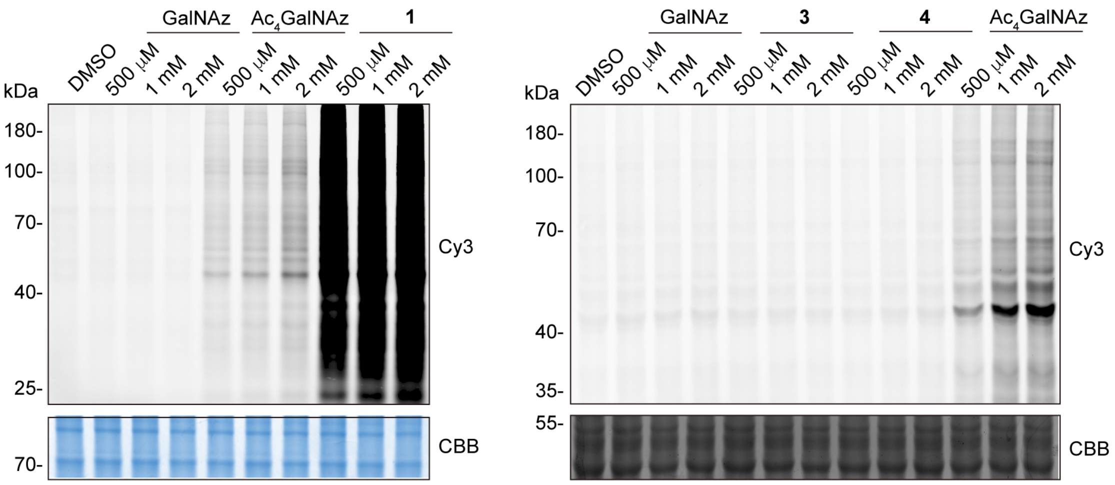

Supplementary Fig. 2 | The gels of Fig. 2a and Fig. 2c shown with a higher contrast. The data shown are from three independent experiments. Source data for figures are provided as a Source Data file.

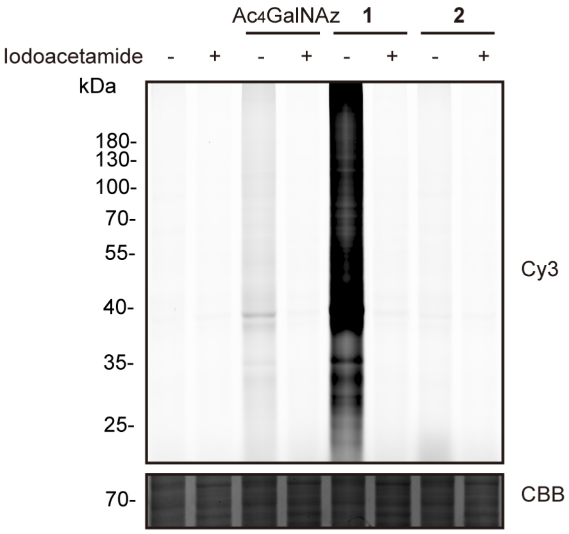

Supplementary Fig. 3 | Blocking of S-glycosylation in cell lysates by pre-treatment with iodoacetamide. In-gel fluorescence scanning showing HeLa cell lysates pre-treated with 25 mM iodoacetamide for 1 h at 37 °C, followed by treatment with 1 mM **1**, **2**, or Ac_4GalNAz for 2 h, and reacted with alkyne-Cy3. Coomassie Brilliant Blue (CBB)-stained gel demonstrates equal loading. The data shown are from three independent experiments. Source data for figures are provided as a Source Data file.

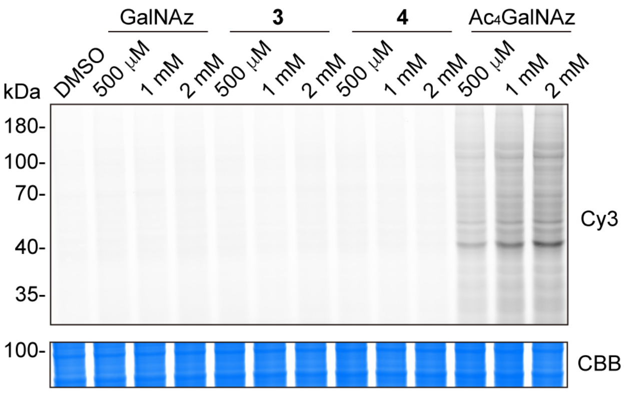

Supplementary Fig. 4 | 1,3-Ac_2GalNAz and 1,3-Pr_2GalNAz do not react with HEK293T lysates. In-gel fluorescence scanning showing HEK293T cell lysates treated with respective unnatural monosaccharides at varied concentrations for 2 h, followed by reaction with alkyne-Cy3. CBB-stained gel demonstrates equal loading. Representative results are shown from three independent experiments. Source data for figures are provided as a Source Data file.

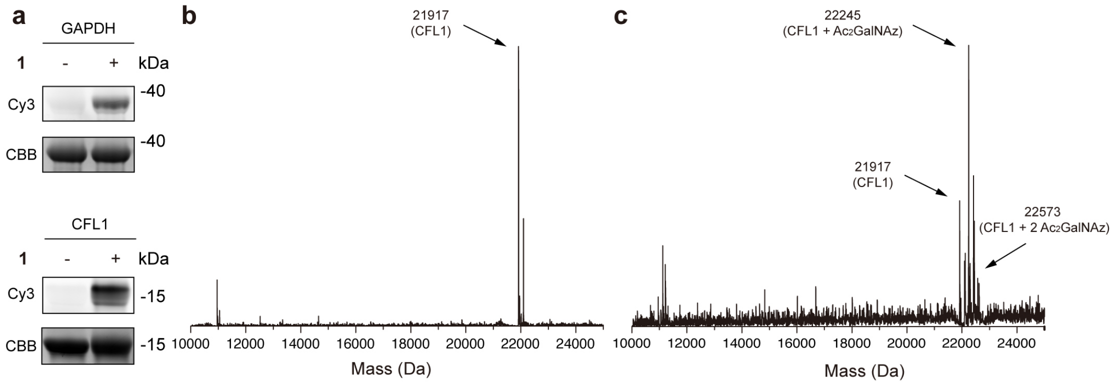

Supplementary Fig. 5 | Reaction of GAPDH and CFL1 with 3,4,6-Ac_3GalNAz (**1**). **a**, In-gel fluorescence scanning showing purified GAPDH and CFL1 treated with 1 mM **1** for 2 h in PBS, followed by reaction with alkyne-Cy3. **b**, Decovoluted mass spectra of CFL1 (left) and CFL1 reacted with **1** (right). The 328-Da and 656-Da mass shift indicted one or two molecules of Ac_2GalNAz were added to one CFL1 protein. Source data for figures **a** are provided as a Source Data file.

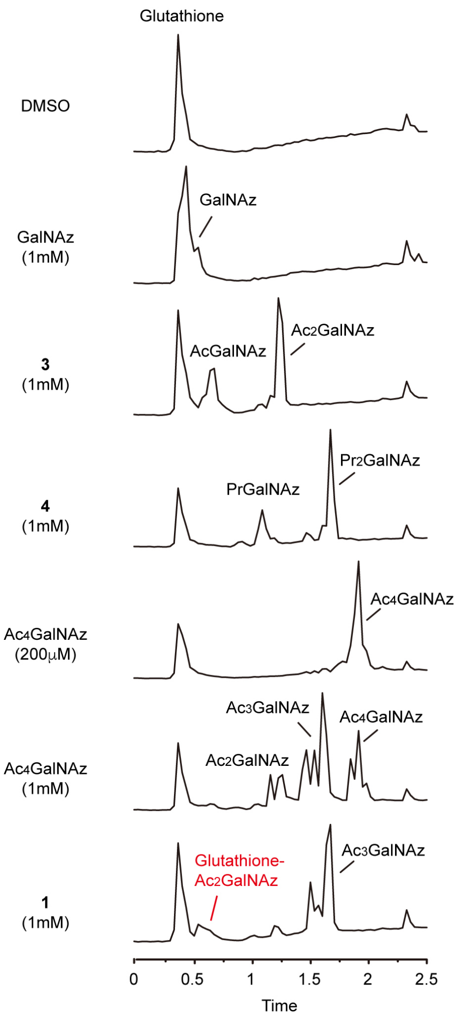

Supplementary Fig. 6 | LC-MS analysis showing reactions between 5 mM glutathione and respective unnatural sugars. 5 mM glutathione was incubated with different unnatural monosaccharides at indicated concentrations for 48 h, followed by detecting the final products with LC-MS.

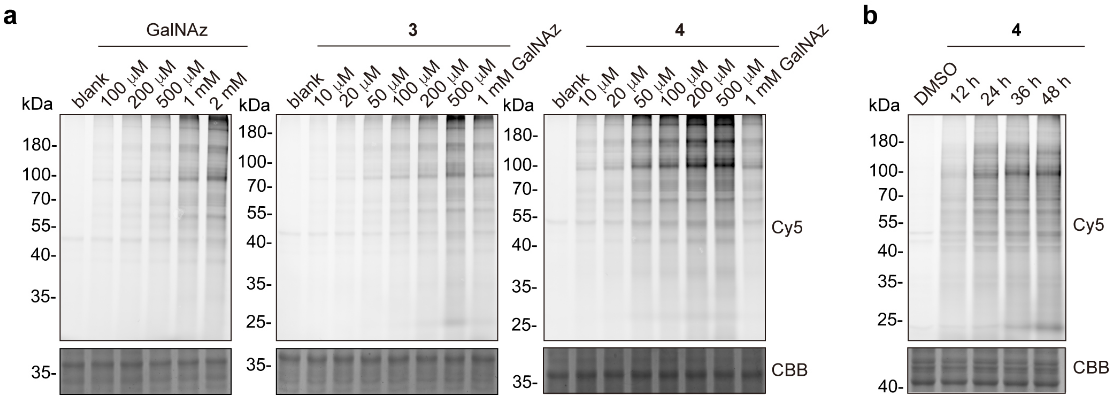

Supplementary Fig. 7 | The dose- and time- dependence of 1,3-Pr_2GalNAz labeling in HeLa cells. **a**, In-gel fluorescence scanning showing HeLa cells incubated with respective unnatural sugars at varied concentrations for 48 h, followed by reaction with alkyne-Cy5. **b**, In-gel fluorescence scanning showing HeLa cells incubated with 100 μM Pr_2GalNAz (**4**) for up to 48 h, followed by reaction with alkyne-Cy5. CBB-stained gel in **a** and **b** demonstrate equal loading. Representative results are shown from three independent experiments. Source data for figures **a** and **b** are provided as a Source Data file.

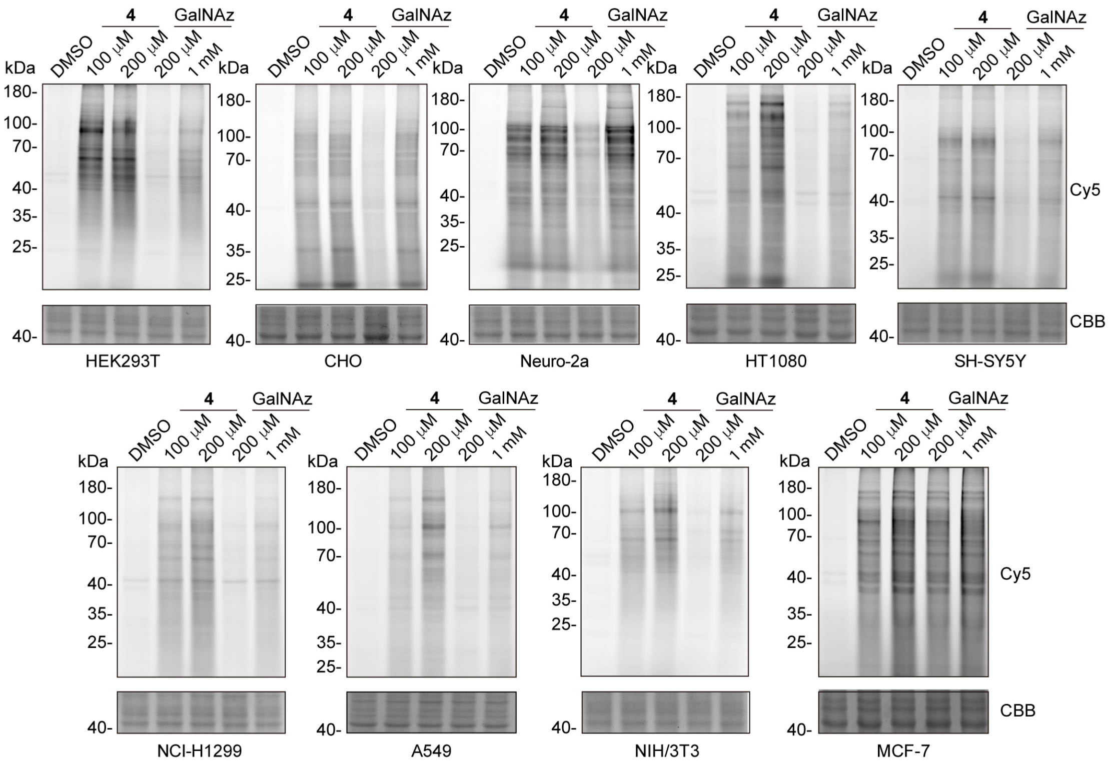

Supplementary Fig. 8 | Comparison of 1,3-Pr_2GalNAz and GalNAz in various cell lines. In-gel fluorescence scanning showing cells incubated with Pr_2GalNAz (**4**) or GalNAz at varied concentrations for 48 h, followed by reaction with alkyne-Cy5. CBB-stained gels demonstrate equal loading. Representative results are shown from three independent experiments. Source data for figures are provided as a Source Data file.

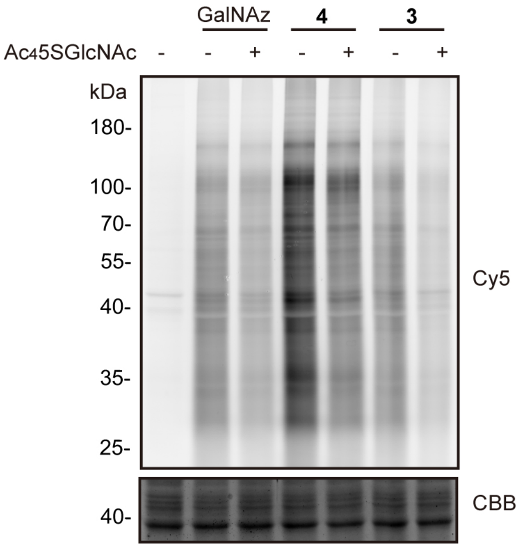

Supplementary Fig. 9 | The OGT-dependent labeling of GalNAz, 1,3-Ac_2GalNAz and 1,3-Pr_2GalNAz in HeLa cells. In-gel fluorescence scanning showing HeLa cells incubated with 1 mM GalNAz, 200 μM 1,3-Ac_2GalNAz (**3**) or 100 μM 1,3-Pr_2GalNAz (**4**) for 48 h, during which the cells were co-treated with 50 μM $Ac_4$5SGlcNAc or vehicle, followed by reaction with alkyne-Cy5. CBB-stained gel demonstrates equal loading. The data shown are from three independent experiments. Source data for figures are provided as a Source Data file.

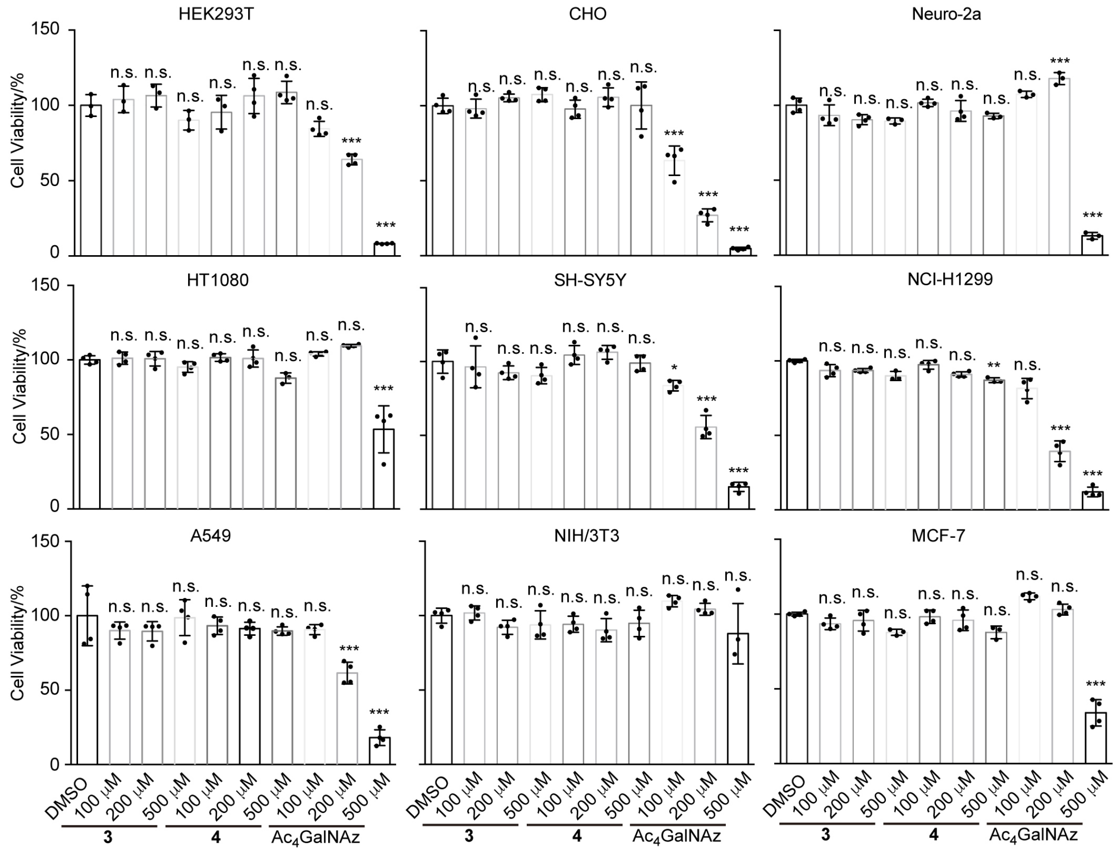

Supplementary Fig. 10 | Cytotoxicity of unnatural monosaccharides. Cell counting assay by CCK-8 kit showing viability of various cells incubated with respective unnatural monosaccharides at varied concentrations for 48 h. Error bars represent mean ± s.d.. Results are from at least three independent experiments. *P < 0.05, **P < 0.01, ***P < 0.001, n.s., not significant (one-way ANOVA). Source data for figures are provided as a Source Data file.

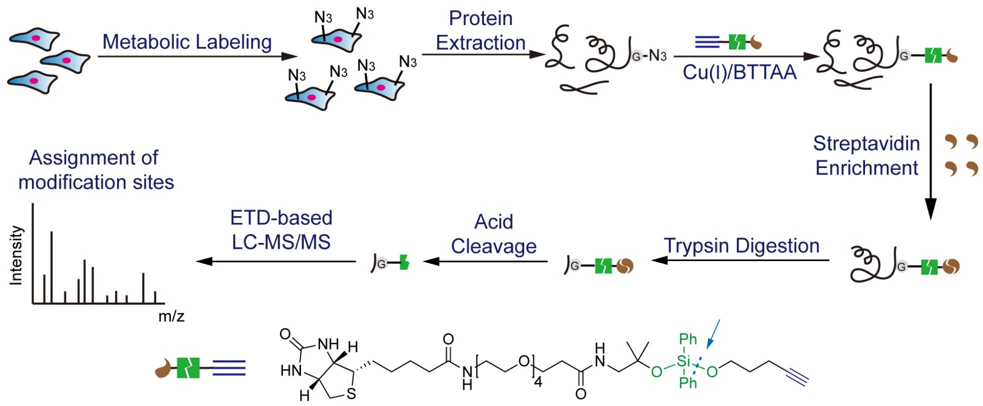

Supplementary Fig. 11 | Workflow for O-GlcNAcylation sites identification. HeLa cells were incubated with $Ac_2GalNAz$ or $Pr_2GalNAz$ for 48 h. The cell lysates were reacted with alkyne-AC-biotin, captured with streptavidin beads, and digested by trypsin. The beads were then incubated in 2% formic acid for totally 2 h to release the O-GlcNAcylated peptides, which were subjected to analysis by ETD-based LC-MS/MS. The blue arrow and dashed lines showed the acid cleavage site.

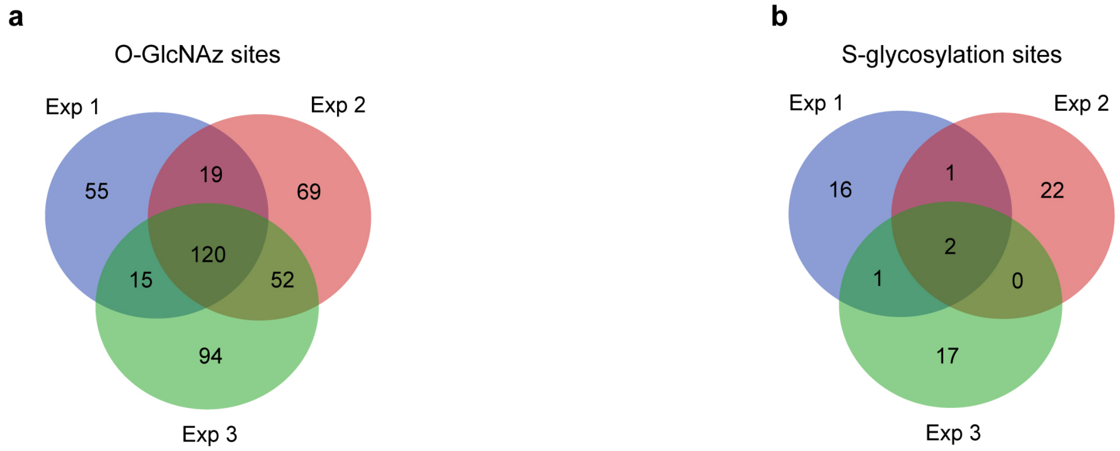

Supplementary Fig. 12 | Large-scale identification of O-GlcNAcylation sites in HeLa cells treated with Ac_2GalNAz. **a,** Overlap of O-GlcNAz sites identified in three biological replicates. HeLa cells were treated with 200 μM 1,3-Ac_2GalNAz (**3**), followed by reaction with alkyne-AC-biotin and LC-MS/MS analysis. **b,** Overlap of S-glycosylation sites identified in three biological replicates. Those identified in at least two replicates were categorized as high-confidence sites.

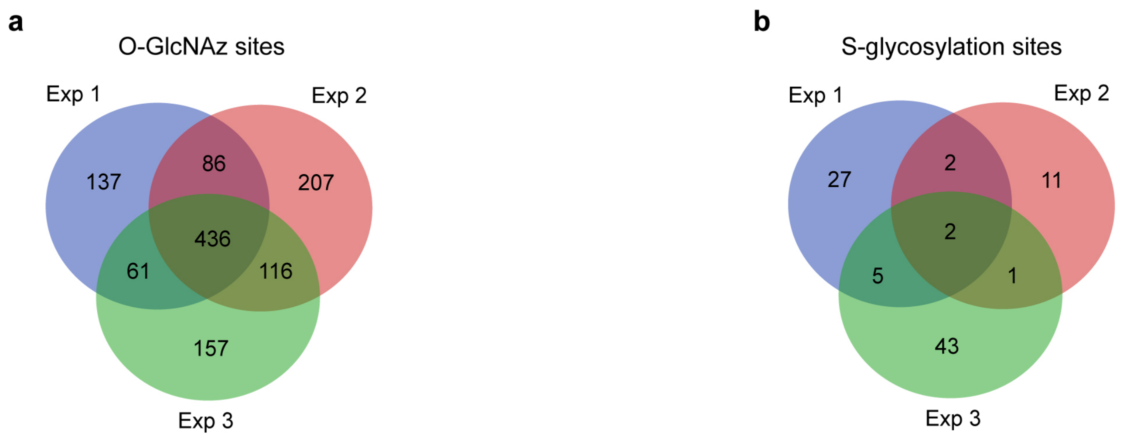

Supplementary Fig. 13 | Large-scale identification of O-GlcNAcylation sites in HeLa cells treated with Pr_2GalNAz. **a,** Overlap of O-GlcNAz sites identified in three biological replicates. HeLa cells were treated with 100 μM 1,3-Pr_2GalNAz (**4**), followed by reaction with alkyne-AC-biotin and LC-MS/MS analysis. **b,** Overlap of S-glycosylation sites identified in three biological replicates. Those identified in at least two replicates were categorized as high-confidence sites.

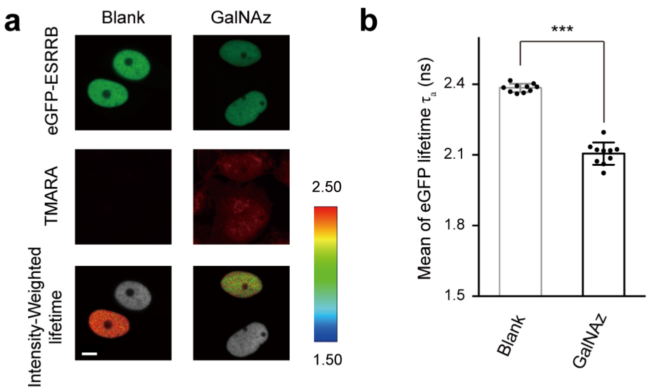

Supplementary Fig. 14 | FLIM-FRET imaging of ESRRB O-GlcNAcylation. **a,** Intensity-weighted EGFP lifetime images showing EGFP-ESRRB overexpressing HeLa cells incubated with vehicle or 1mM GalNAz for 48 h, followed by reaction with alkyne-TMARA. Decreased in EGFP lifetime indicates intramolecular FRET between TMARA-labeled O-GlcNAc and EGFP on ESRRB. Scale bar, 6 µm. **b**, Bar graph showing statistical analysis of the average lifetime of a single cell (τ_a). The τ_a value was calculated from the τ distribution of individual cells. Error bars represent mean ± s.d.. Results are from a total of 10 cells collected from three independent experiments. ***P <0.001 (Student's t-test). Source data for figure **b** are provided as a Source Data file.

Rat_ESRRB	IKTEPSSP**SS**GIDALSHHSPS
Mouse_ESRRB	IKTEPSSP**SS**GIDALSHHSPS
Human_ESRRB	IKTEPSSP**SS**GIDALSHHSPS

Supplementary Fig. 15 | Alignment of O-GlcNAc-containing peptides of ESRRB in human, rat and mouse.

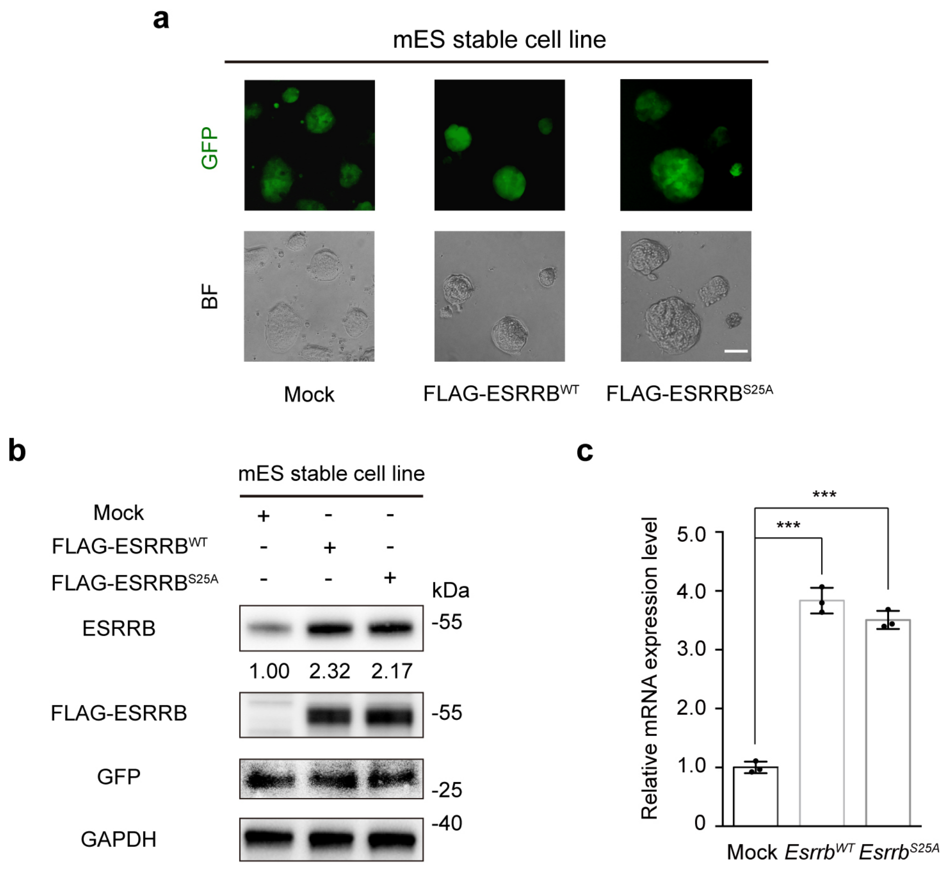

Supplementary Fig. 16 | Generation of mESCs stably expressing FLAG-ESRRB. **a,** Fluorescent and bright-filed images of mESCs expressing pCDH-EF1-T2A-copGFP (Mock), pCDH-EF1-FLAG-ESRRB^{WT}-T2A-copGFP and pCDH-EF1-FLAG-ESRRB^{S25A}-T2A-copGFP. Scale bar: 50 μm. **b,** Immunoblots showing the expression of Mock, FLAG-ESRRB^{WT} and FLAG-ESRRB^{S25A}. The anti-GAPDH gel demonstrates comparable loading. Representative results are shown from three independent experiments in **b**. **c**, Relative mRNA level of ESRRB in mESCs stably expressing Mock, FLAG-ESRRB^{WT}, or FLAG-ESRRB^{S25A} by qRT-PCR. Error bars represent mean ± s.d.. ***P < 0.001 (one-way ANOVA). Source data for figures **b** and **c** are provided as a Source Data file.

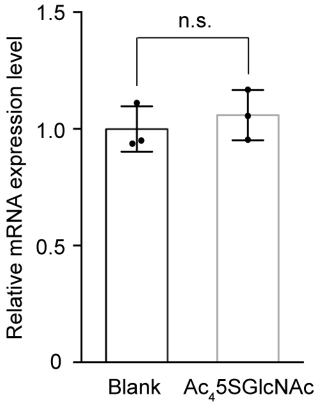

Supplementary Fig. 17 | Relative mRNA level of ESRRB. mESCs were treated with DMSO or 50 μM $Ac_4$5SGlcNAc for 48 h, followed by qRT-PCR analysis of ESRRB mRNA level. Error bars represent mean ± s.d.. Results are from three independent experiments. n.s., not significant (Student's t-test). Source data for figures are provided as a Source Data file.

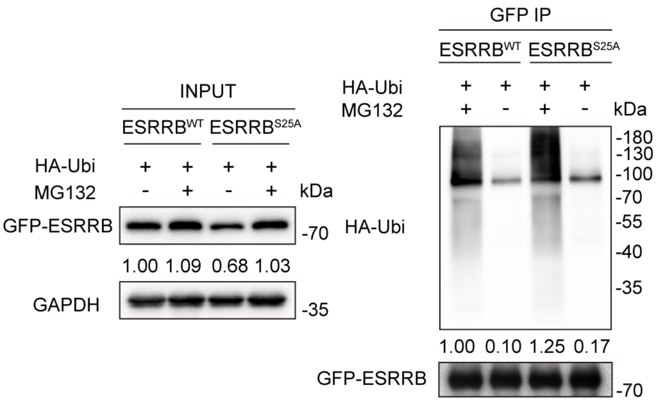

Supplementary Fig. 18 | O-GlcNAcylation of Ser 25 regulates ESRRB stability through ubiquitin-mediated pathway. HEK293T cells were co-transfected with the plasmids of HA-UBIQUITIN and EGFP-ESRRB^{WT} (or EGFP-ESRRB^{S25A}). Then the cell samples were treated with 10 μM MG132 for 10 h before harvest, and the ubiquitination of immunoprecipitated EGFP-ESRRB^{WT} or EGFP-ESRRB^{S25A} was detected by anti-HA. Anti-GFP or anti-GAPDH indicated the loading control. The relative protein level was calculated as the ratio according to the band intensity and the loading control. The ratio was then normalized to the level of EGFP-ESRRB^{WT} at 0 h. The data shown are from three independent experiments. Source data for figures are provided as a Source Data file.

Ensembl ID	Gene name	Log_2(WT/S25A)	$-Log_{10}$(q value)	ESRRB target
ENSMUSG00000027070	Lrp2	0.83367453	3.17E-48	Yes
ENSMUSG00000000730	Dnmt3l	0.792871913	1.59E-33	Yes
ENSMUSG00000026726	Cubn	0.646030148	3.19E-32	Yes
ENSMUSG00000034777	Vax2	-0.938090706	4.52E-30	Yes
ENSMUSG00000020077	Srgn	0.59942329	5.18E-16	Yes
ENSMUSG00000027082	Tfpi	0.598839138	1.65E-15	Yes
ENSMUSG00000078952	Lncenc1	0.87254283	1.01E-13	No
ENSMUSG00000031216	Stard8	0.667838415	1.04E-13	Yes
ENSMUSG00000085794	Vax2os	-0.866750934	5.49E-13	No
ENSMUSG00000069045	Ddx3y	0.899929137	5.60E-13	No
ENSMUSG00000032492	Pth1r	0.785572788	2.27E-12	Yes
ENSMUSG00000022237	Ankrd33b	0.641304946	1.29E-11	Yes
ENSMUSG00000050424	Pnma5	0.906122151	1.11E-10	No
ENSMUSG00000028195	Ccn1	0.771249819	4.61E-10	No
ENSMUSG00000052353	Cemip	0.87148871	5.88E-09	No
ENSMUSG00000031410	Nxf7	0.811675788	6.74E-08	No
ENSMUSG00000000805	Car4	0.978487189	9.53E-08	Yes
ENSMUSG00000116295	Gm32885	1.447450666	1.66E-07	No
ENSMUSG00000033491	Prss35	0.667136641	1.84E-07	Yes
ENSMUSG00000057836	Xlr3a	1.422464994	2.20E-07	No
ENSMUSG00000017607	Tns4	1.146996063	4.88E-07	Yes
ENSMUSG00000000031	H19	0.696822586	6.65E-07	Yes
ENSMUSG00000038415	Foxq1	0.621584317	2.95E-06	Yes
ENSMUSG00000021456	Fbp2	0.637620195	3.09E-06	Yes
ENSMUSG00000072774	Zfp951	1.1662626	3.60E-06	No
ENSMUSG00000027985	Lef1	1.017751369	4.98E-06	Yes
ENSMUSG00000062991	Nrg1	0.616740472	3.79E-05	Yes
ENSMUSG00000115318	Gm49089	1.366636902	3.93E-05	No
ENSMUSG00000024803	Ankrd1	1.364995786	4.66E-05	Yes
ENSMUSG00000020679	Hnf1b	0.668670684	0.000112628	Yes
ENSMUSG00000040152	Thbs1	1.316864055	0.000131377	Yes
ENSMUSG00000059325	Hopx	0.749892901	0.000159453	Yes
ENSMUSG00000039385	Cdh6	0.954018786	0.000178418	Yes
ENSMUSG00000068457	Uty	0.898422734	0.000189879	No
ENSMUSG00000021848	Otx2	1.151958666	0.000247168	Yes
ENSMUSG00000023224	Serping1	0.597902875	0.000266936	Yes
ENSMUSG00000032548	Slco2a1	0.667030373	0.000281552	Yes

Notes: Genes with increased or decreased expression (fold change cutoff 1.5 and q value < 0.05)

Supplementary Fig. 19 | Genes with increased or decreased expression between mESCs stably expressing FLAG-ESRRB^{WT} and FLAG-ESRRB^{S25A} by RNA sequencing from three biological replicates.

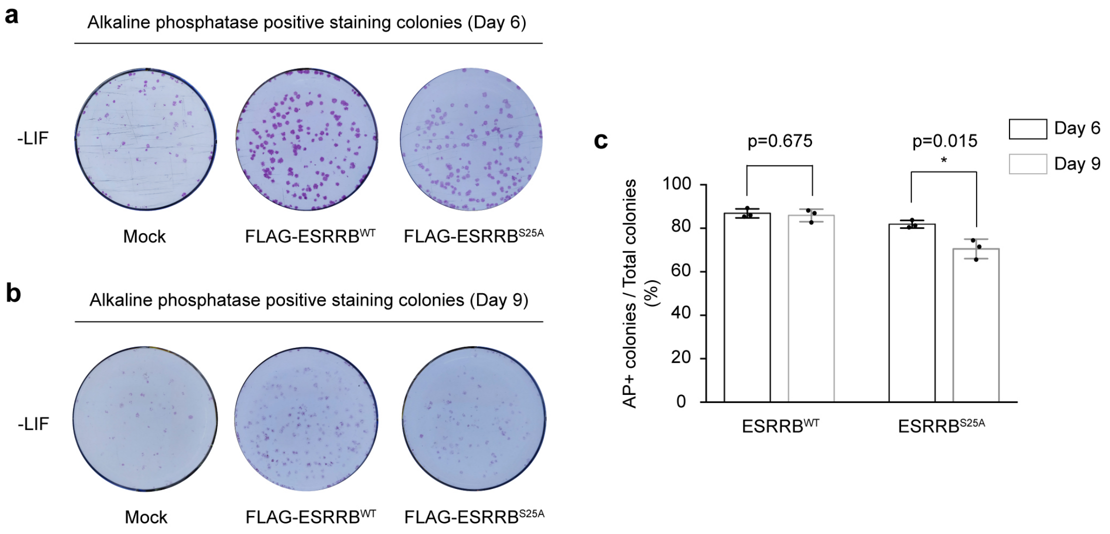

Supplementary Fig. 20 | AP-staining showing undifferentiated states of mESCs expressing vehicle, FALG-ESRRB, or FLAG-ESRRB^{S25A}. **a,** Representative AP-staining of mESC cultured in serum medium without LIF for 6 d. **b,** Representative AP-staining of mESC cultured in serum medium with no LIF for 9 d. **c,** Bar graphs showing the proportion of AP positive colonies in total mESCs cultured in serum medium withdraw LIF for 6 d and 9 d. Error bars represent mean ± s.d.. Results are from three independent experiments. *$P < 0.05$ (Student's t-test). Source data for figure **c** are provided as a Source Data file.

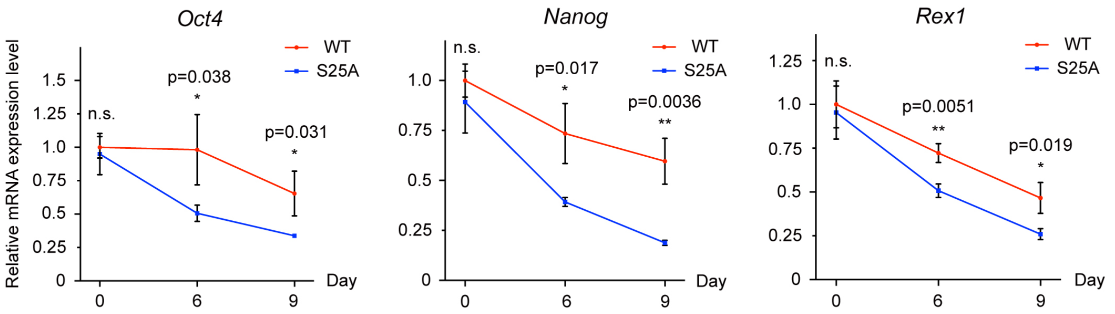

Supplementary Fig. 21 | Quantitative RT-PCR analysis of pluripotent genes in mESCs stably expressing ESRRB^{WT} and ESRRB^{S25A} cultured with or without LIF. FLAG-ESRRB expressing mESCs were cultured in the serum medium with LIF (Day 0) or without LIF for 6 day and 9 d. Bar graphs showing the relative mRNA expression of mESC marker genes, including *Oct4*, *Nanog* and *Rex1*. Error bars represent mean ± s.d.. Results are from three independent experiments. *P < 0.05, **P < 0.01 (Student's t-test). Source data for figures are provided as a Source Data file.

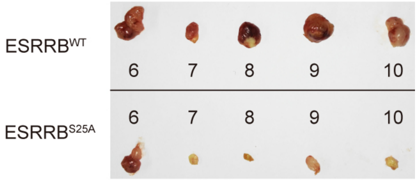

Supplementary Fig. 22 | Teratoma formation by mESCs expressing FLAG-ESRRB^{WT} and FLAG-ESRRB^{S25A} in the other 5 immuno-deficient nude-mice. These mice injected for teratoma formation were numbered from 6 to 10.

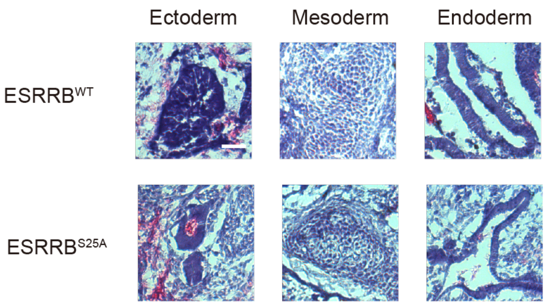

Supplementary Fig. 23 | Representative of three germ layers (ectoderm, mesoderm and endoderm) of teratomas in immunodeficient nude-mice. Teratomas formed from mESCs expressing FLAG-ESRRB^{WT} and FLAG-ESRRB^{S25A} were fixed with 4% paraformaldehyde, dehydrated with 30% (w/v) sucrose, and sliced into 10 μM sections. Sections were stained with hematoxylin and eosin for imaging. Scale bar: 50 μm.

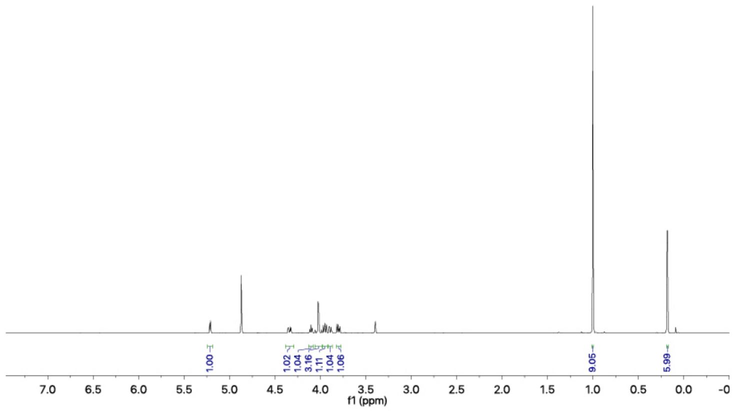

Supplementary Fig. 24 | ^{1}H-NMR spectrum of (**7**)

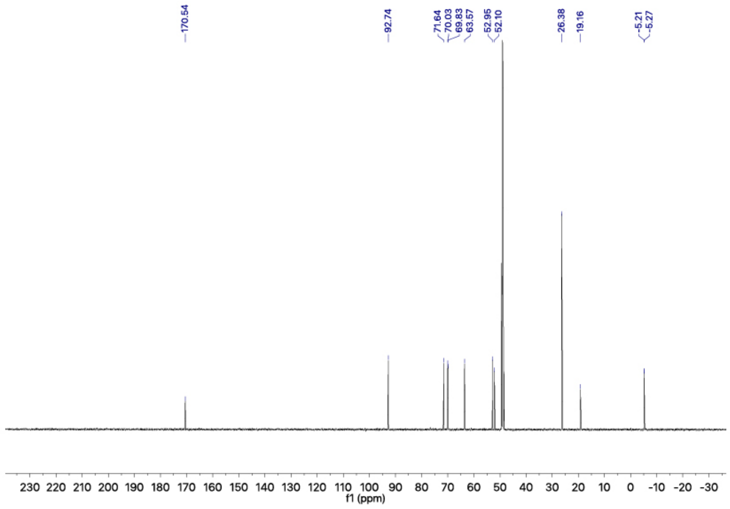

Supplementary Fig. 25 | ^{13}C-NMR spectrum of (**7**)

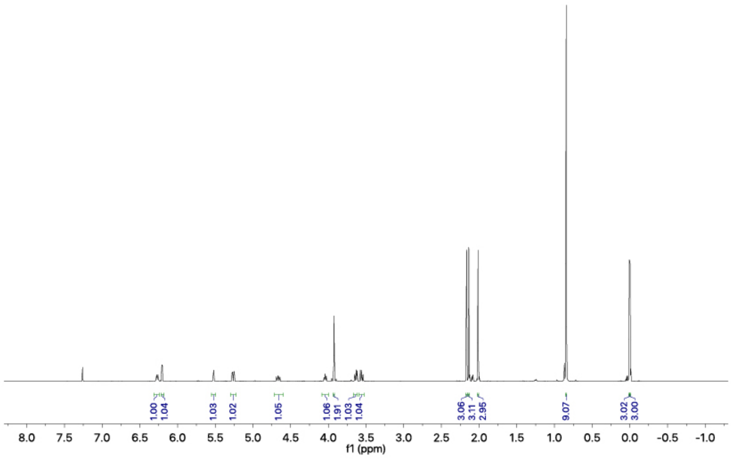

Supplementary Fig. 26 | ^{1}H-NMR spectrum of (**8**)

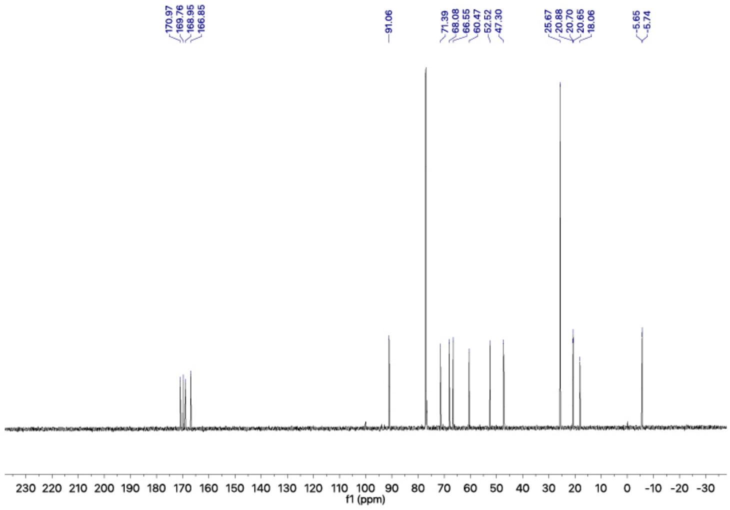

Supplementary Fig. 27 | ^{13}C-NMR spectrum of (**8**)

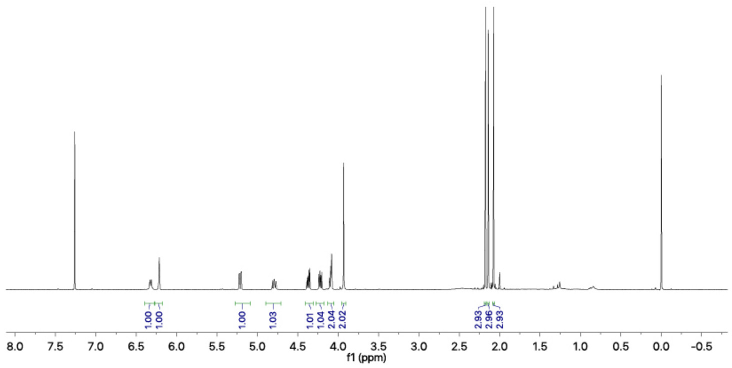

Supplementary Fig. 28 | ^{1}H-NMR spectrum of (**2**)

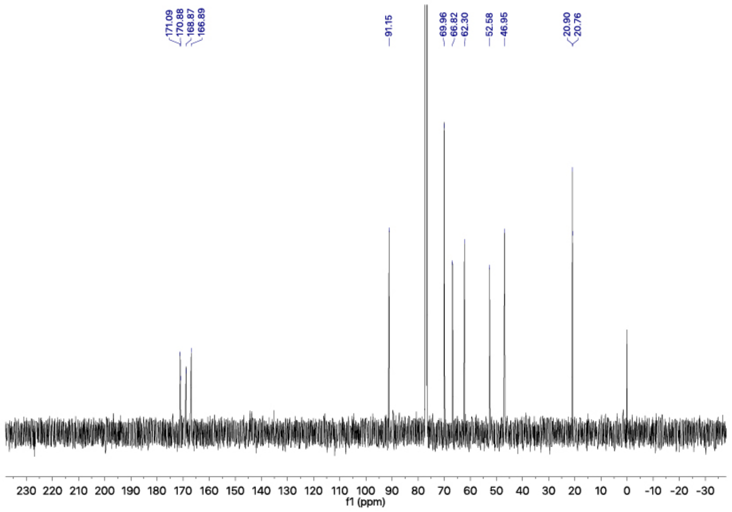

Supplementary Fig. 29 | ^{13}C-NMR spectrum of (**2**)

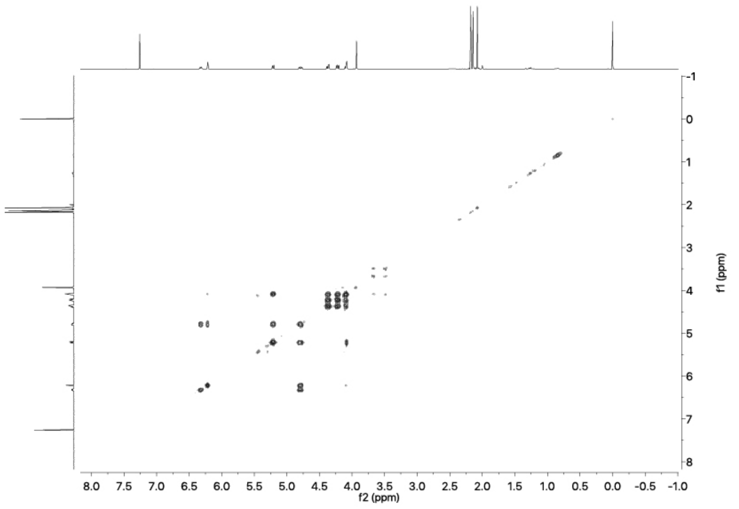

Supplementary Fig. 30 | COSY-NMR spectrum of (**2**)

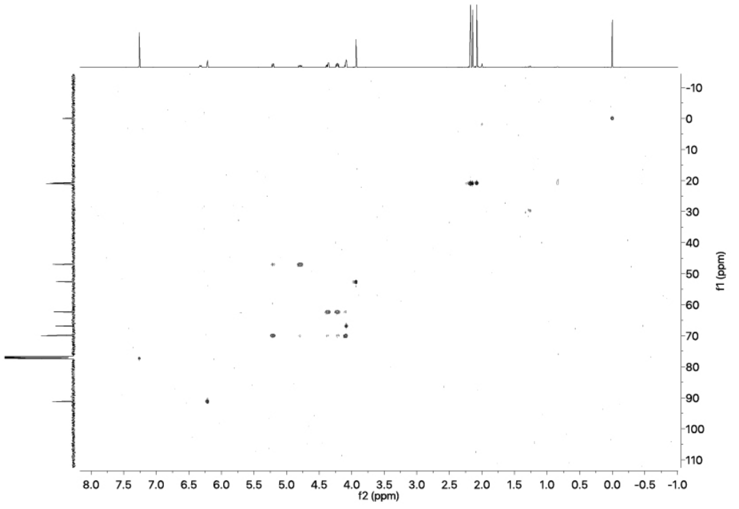

Supplementary Fig. 31 | HSQC-NMR spectrum of (**2**)

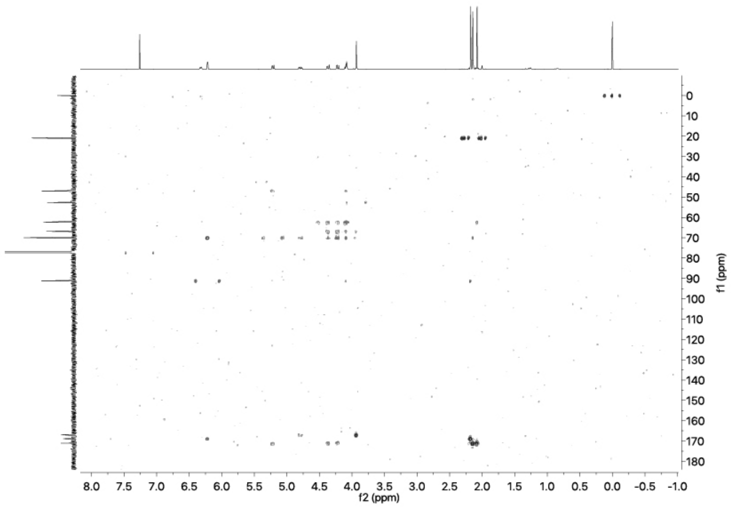

Supplementary Fig. 32 | HMBC-NMR spectrum of (**2**)

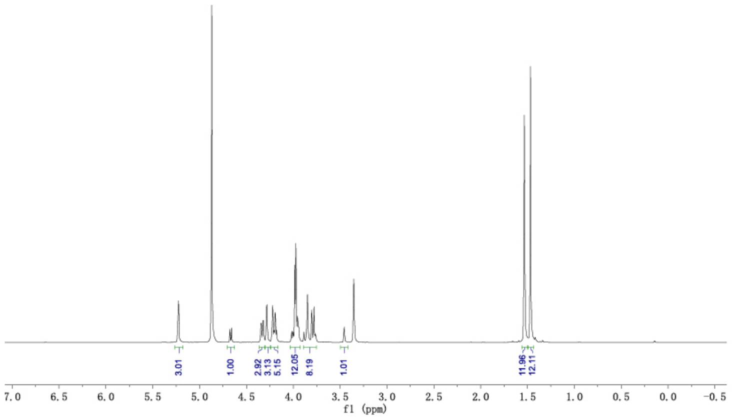

Supplementary Fig. 33 | ^{1}H-NMR spectrum of (**9**)

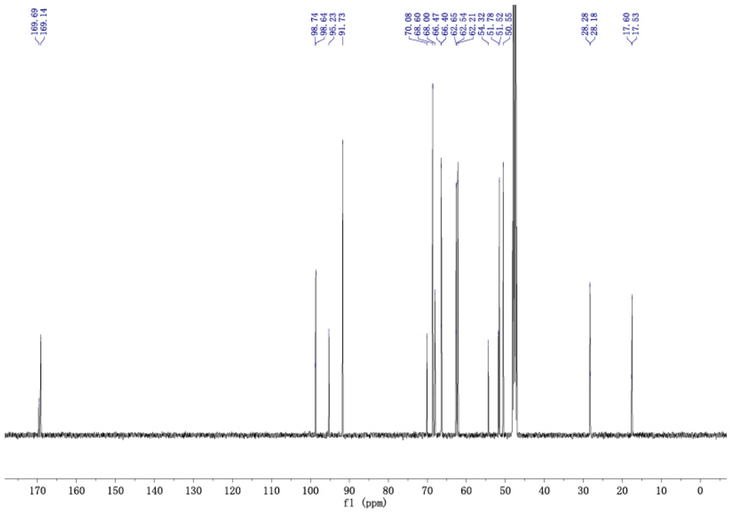

Supplementary Fig. 34 | ^{13}C-NMR spectrum of (**9**)

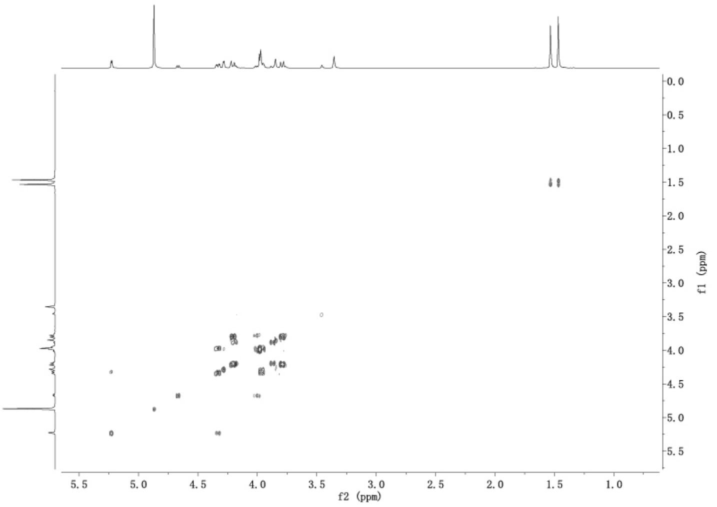

Supplementary Fig. 35 | COSY-NMR spectrum of (**9**)

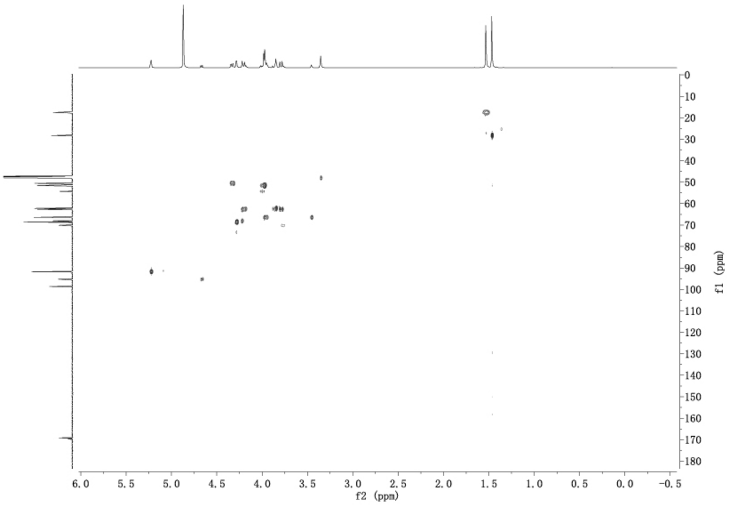

Supplementary Fig. 36 | HSQC-NMR spectrum of (**9**)

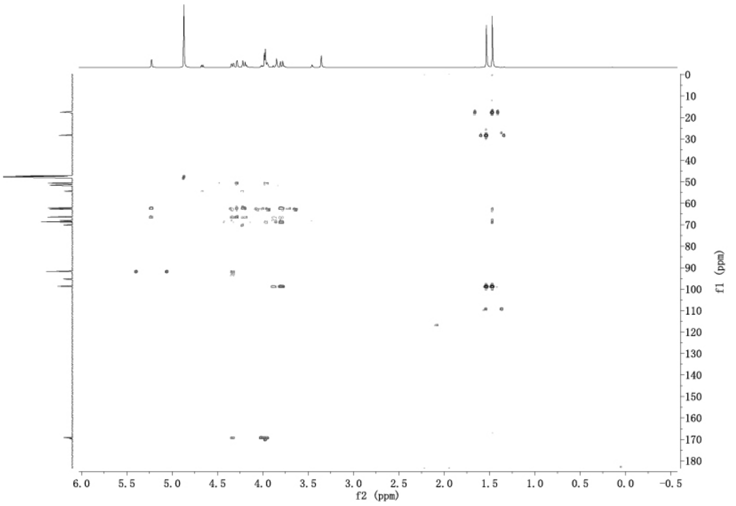

Supplementary Fig. 37 | HMBC-NMR spectrum of (**9**)

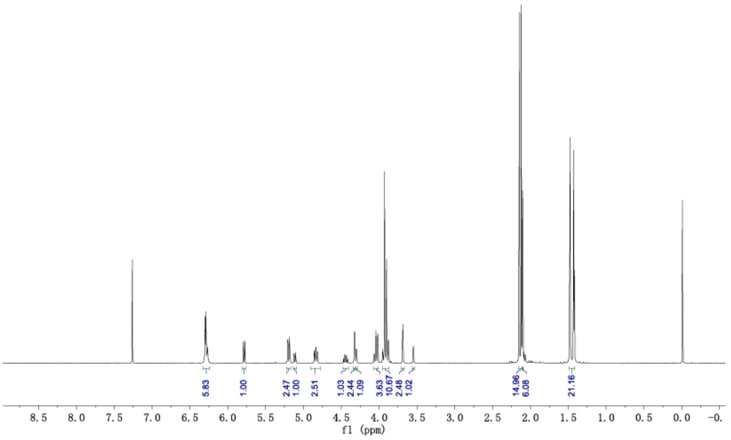

Supplementary Fig. 38 | ^{1}H-NMR spectrum of (**10**)

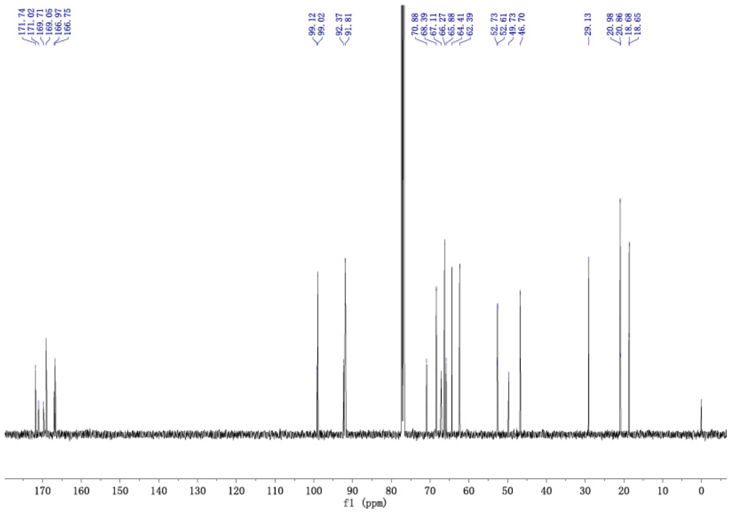

Supplementary Fig. 39 | ^{13}C-NMR spectrum of (**10**)

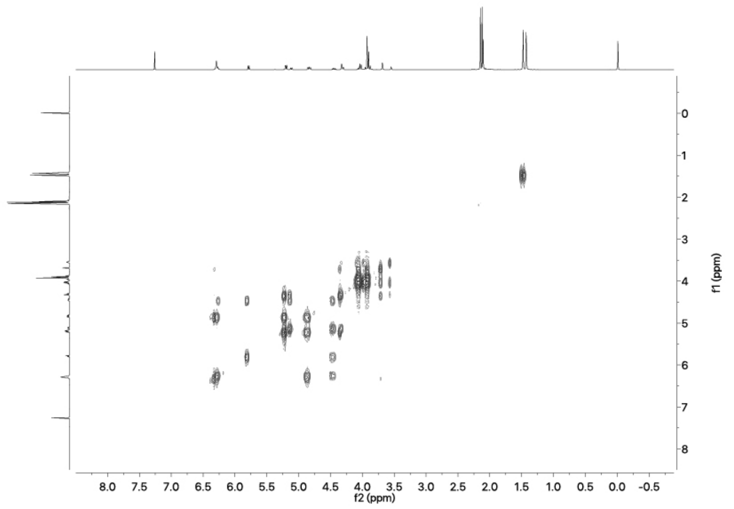

Supplementary Fig. 40 | COSY-NMR spectrum of (**10**)

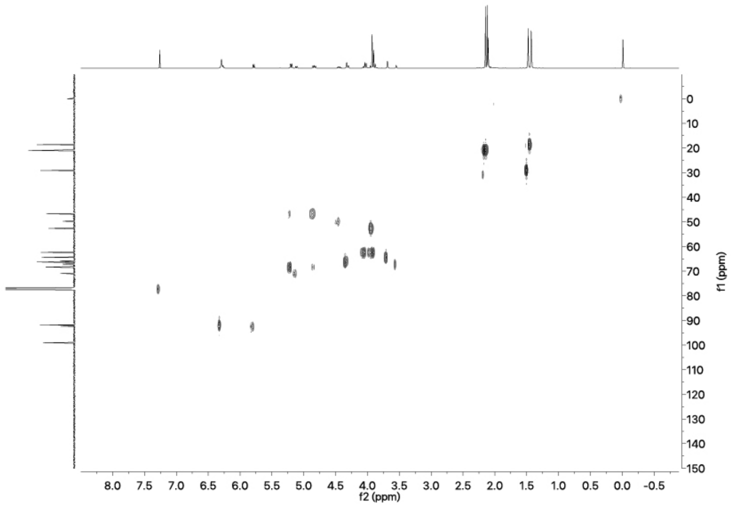

Supplementary Fig. 41 | HSQC-NMR spectrum of (**10**)

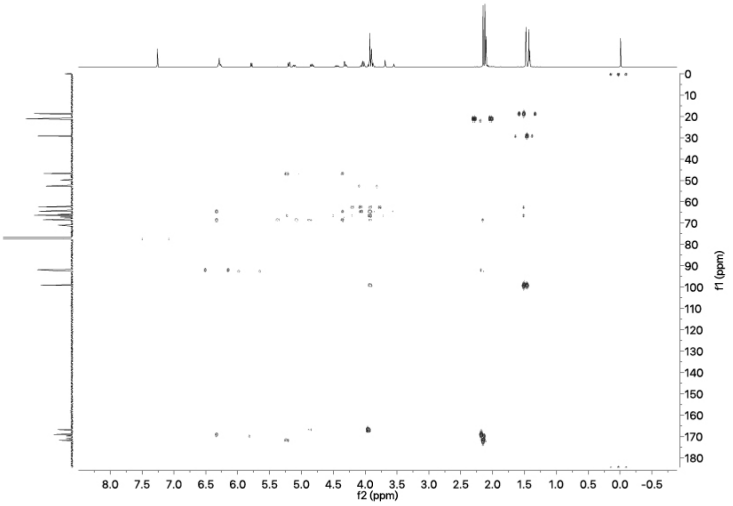

Supplementary Fig. 42 | HMBC-NMR spectrum of (**10**)

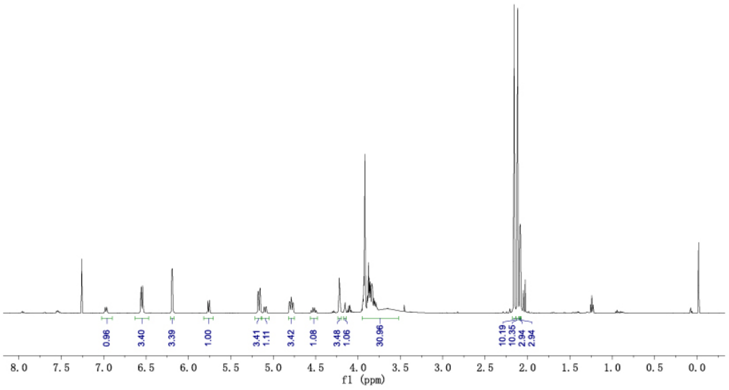

Supplementary Fig. 43 | ^{1}H-NMR spectrum of (**3**)

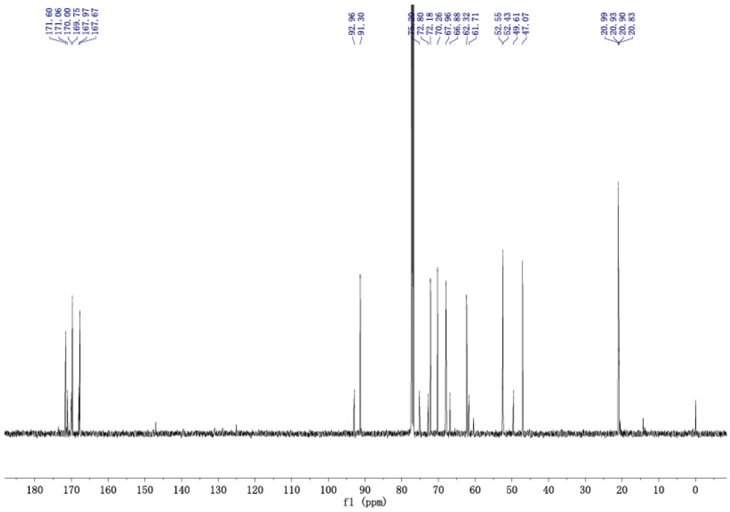

Supplementary Fig. 44 | ^{13}C-NMR spectrum of (**3**)

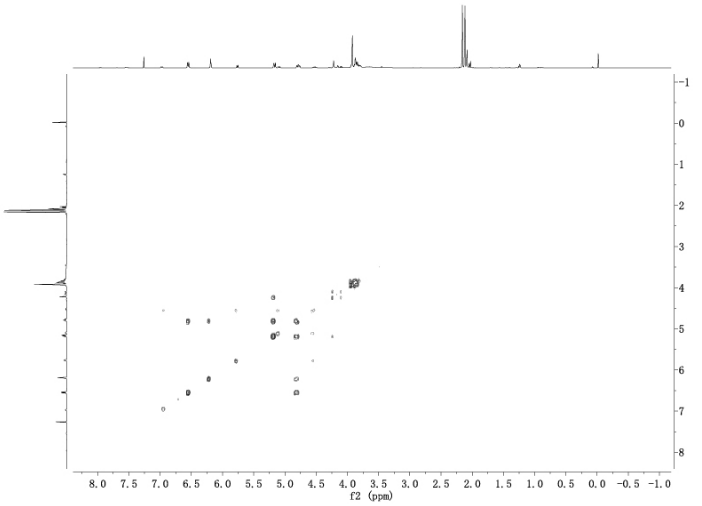

Supplementary Fig. 45 | COSY-NMR spectrum of (**3**)

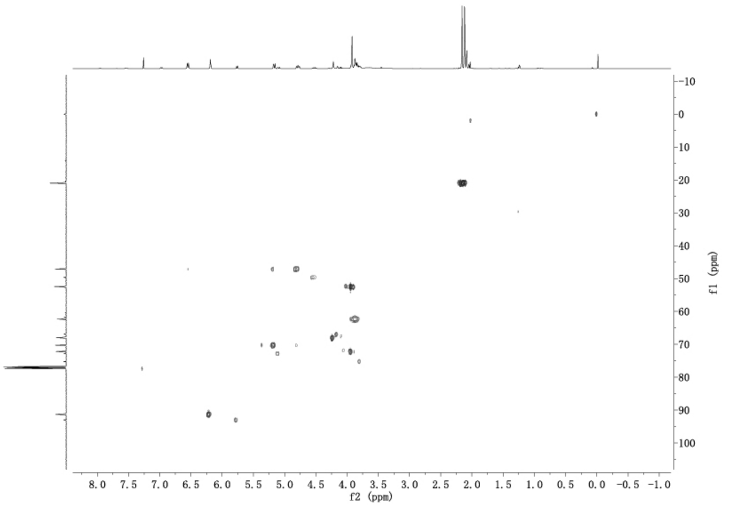

Supplementary Fig. 46 丨 HSQC-NMR spectrum of (**3**)

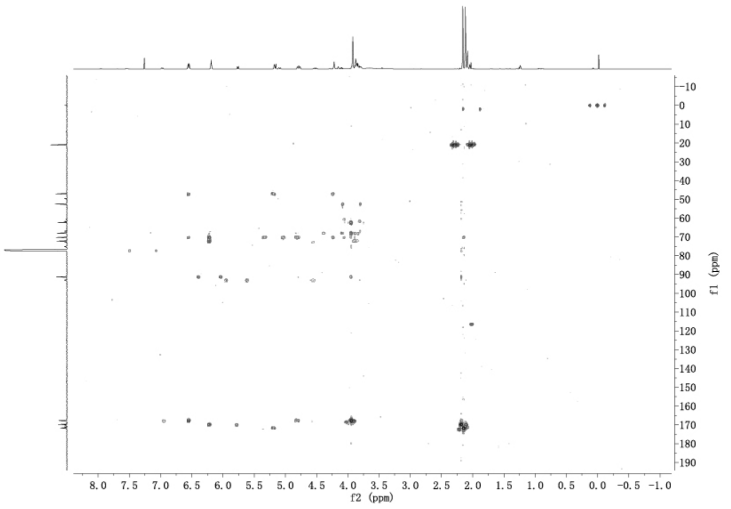

Supplementary Fig. 47 丨 HMBC-NMR spectrum of (**3**)

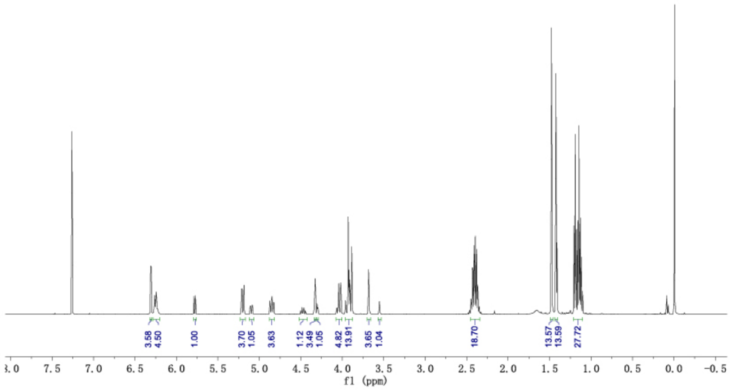

Supplementary Fig. 48 | ^{1}H-NMR spectrum of (**11**)

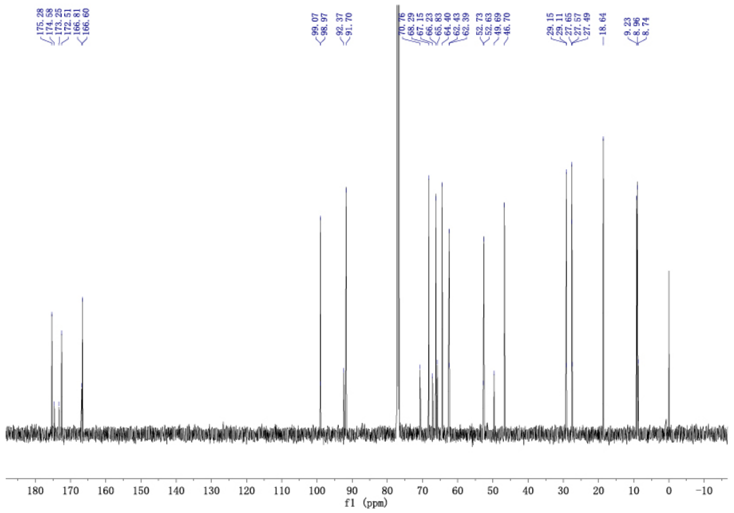

Supplementary Fig. 49 | ^{13}C-NMR spectrum of (**11**)

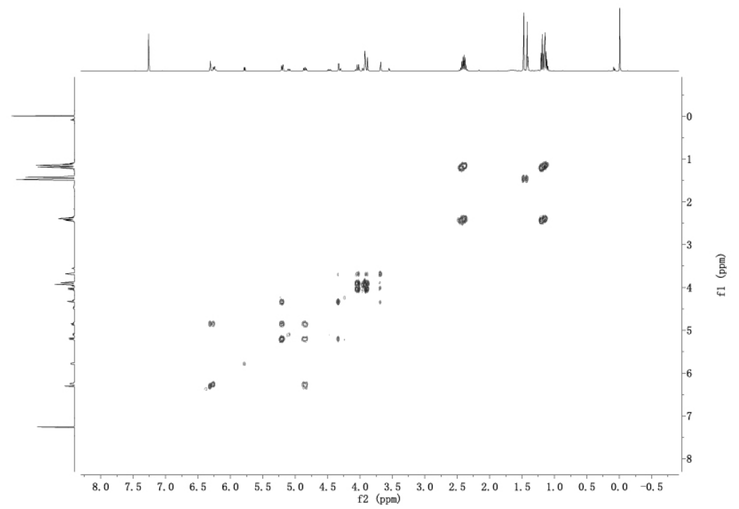

Supplementary Fig. 50 | COSY-NMR spectrum of (**11**)

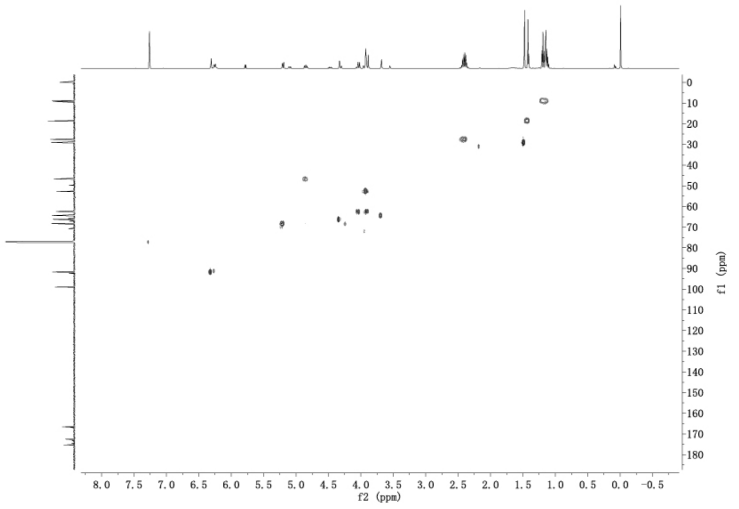

Supplementary Fig. 51 | HSQC-NMR spectrum of (**11**)

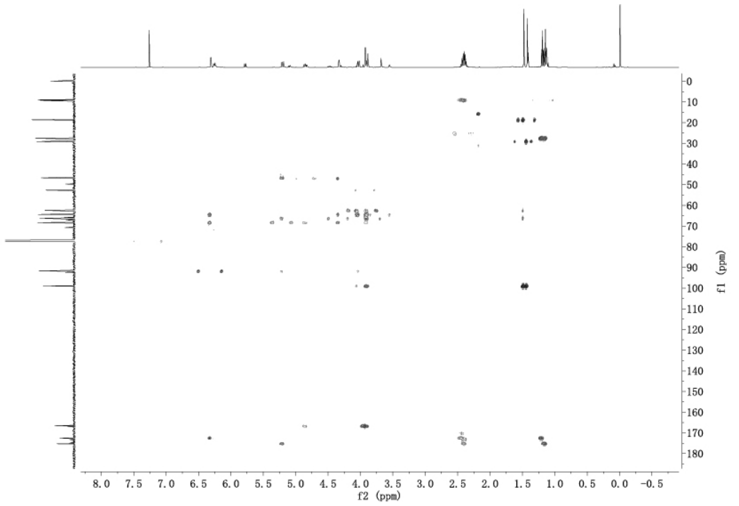

Supplementary Fig. 52 | HMBC-NMR spectrum of (**11**)

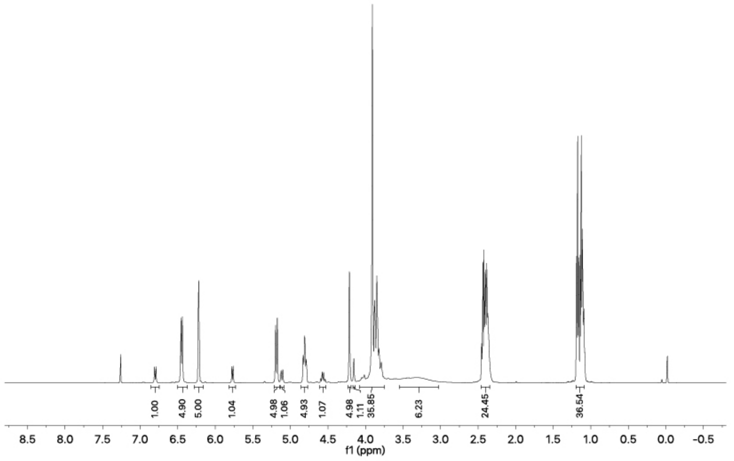

Supplementary Fig. 53 | ^{1}H-NMR spectrum of (**4**)

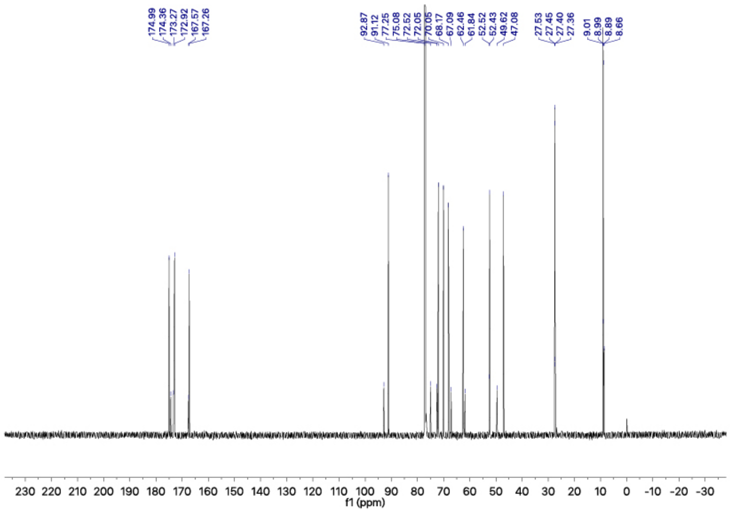

Supplementary Fig. 54 | ^{13}C-NMR spectrum of (**4**)

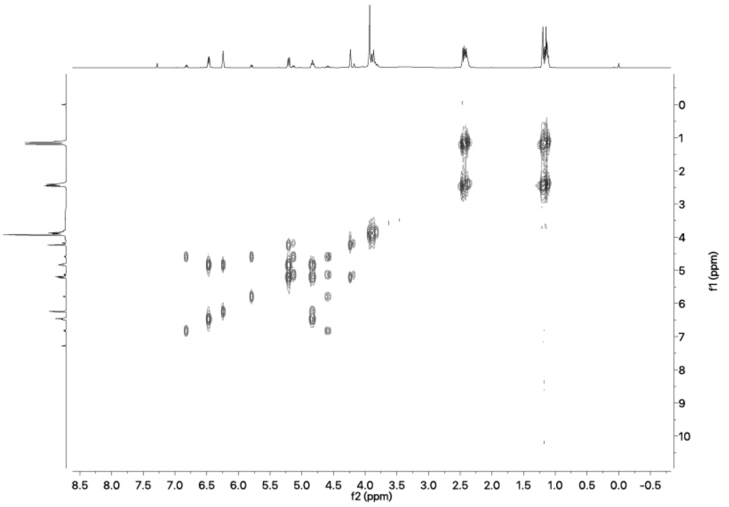

Supplementary Fig. 55 | COSY-NMR spectrum of (**4**)

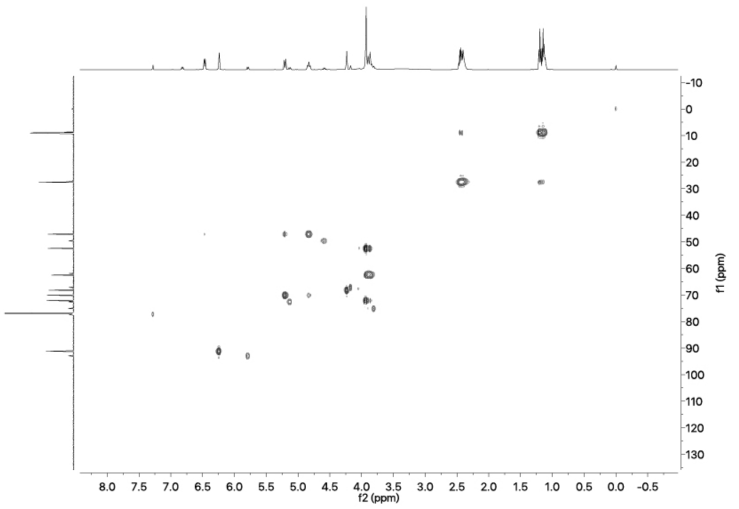

Supplementary Fig. 56 | HSQC-NMR spectrum of (**4**)

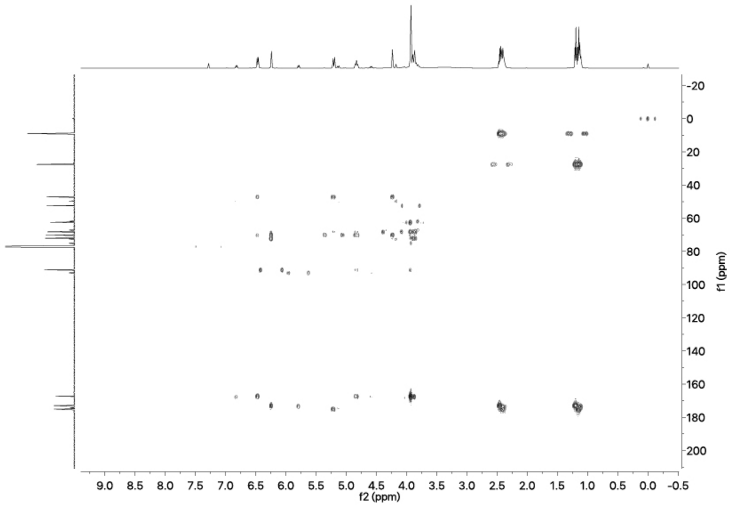

Supplementary Fig. 57 | HMBC-NMR spectrum of (**4**)

Supplementary Note 1

Synthesis of 6-tert-butyldimethylsilyl-*N*-azidoacetylgalactosamine (7)

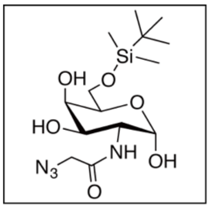

To a solution of GalNAz (470 mg, 1.79 mmol) in 10 mL pydrine was added tert-butyldimethylsilyl chloride (403 mg, 2.67 mmol), and the mixture was stirred at R. T. for 20 h. After removal of pydrine *in vacuo*, the crude product was purified by flash chromatography with MeOH in CH_2Cl_2 gradually from (0 to 5%) to yield product **7** as colorless oil (335 mg, 50%). ^{1}H NMR (500 MHz, CD_3OD) δ 5.21 (d, J=3.5 Hz, 1H), 4.34 (dd, J=10.8, 3.8 Hz, 1H), 4.13-4.08 (m, 1H), 4.06-3.98 (m, 3H), 3.97-3.92 (m, 1H), 3.89 (dd, J=10.8, 3.3 Hz, 1H), 3.83-3.77 (m, 1H), 1.00 (s, 1H), 0.18 (s, 1H), 0.18 (s, 1H). ^{13}C NMR (125 MHz, CD_3OD) δ 170.54, 92.74, 71.64, 70.03, 69.83, 63.57, 52.95, 52.10, 26.38, 19.16, -5.21, -5.27. HRMS (ESI): m/z calculated for $C_{14}H_{29}N_4O_6Si$ $[M+H]^+$ 377.1851, found 377.1850.

Synthesis of 1,3-acetylated-4,6-isopropyidene-*N*-azidoacetylgalactosamine (8)

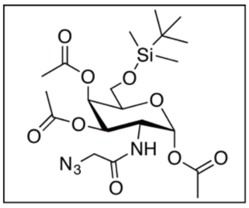

To a stirred solution of compound **7** (335 mg, 0.89 mmol) in 5 mL pydrine at 0 °C was added 0.7 mL acetic anhydride (0.52 mL, 5.3 mmol). The mixture was warmed to R.T. and stirred overnight. The solvent was removed *in vacuo*, and the crude product was further purified by flash chromatography on silica gel, eluted with EA : PE from the volume ratio at 1:100 to 1:1. The compound **8** was provided as white solid (412mg, 92%) after purification. ^{1}H NMR (500 MHz, $CDCl_3$) δ 6.27 (d, J=9.0, 1H), 6.20 (d, J=4.0, 1H), 5.52 (dd, J=3.5, 1.5Hz, 1H), 5.26 (dd, J=11.5, 3.5Hz, 1H), 4.67 (ddd, J=11.6, 9.1, 3.9Hz, 1H), 4.08-4.00 (m, 1H), 3.92 (s, 2H), 3.66-3.61 (m, 1H), 3.59-3.52 (m, 1H), 2.17 (s, 3H), 2.14 (s, 3H), 2.01 (s, 3H), 0.84 (s, 9H), 0.01 (s, 3H), 0.00 (s, 3H). ^{13}C NMR (125 MHz, $CDCl_3$) δ 170.97, 169.76, 168.95, 166.85, 91.06, 71.39, 68.08, 66.55, 60.47, 52.52, 47.30, 25.67, 20.88, 20.70, 20.65, 18.06, -5.65, -5.74. HRMS (ESI): m/z calculated for $C_{20}H_{38}N_5O_9Si$ $[M+NH_4]^+$ 520.2433, found 520.2423, $C_{20}H_{34}N_4O_9SiNa$ $[M+Na]^+$ 520.1987, found 520.1985.

Synthesis of 1,3,6-acetylated -*N*-azidoacetylgalactosamine (2)

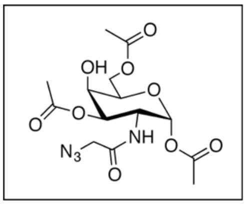

Trifluoroacetic acid (1 mL) was added dropwise to the stirred solution of compound **8** (412 mg, 0.82 mmol) in 10 mL dichloromethane at 0 °C. The reaction mixture was warmed to R.T., followed by stirred for another 1 h. The acid and the solvent were removed under reduced pressure, and the residue was purified by flash chromatography with EA : PE gradually from 0 to 6 : 4 to provide the final product **2** as white solid (125 mg, 39%). ^{1}H NMR (500 MHz, $CDCl_3$) δ 6.32 (d, J=9.5, 1H), 6.22 (d, J=3.5, 1H), 5.21 (dd, J=11.0, 3.0, 1H), 4.79 (ddd, J=11.3, 9.3, 3.8Hz, 1H), 4.41-4.33 (m, 1H), 4.27-4.18 (m, 1H), 4.12-4.05 (m, 1H), 3.93 (s, 2H), 2.18 (s, 3H), 2.14 (s, 1H), 2.08 (s, 1H). ^{13}C NMR (125 MHz, $CDCl_3$) δ 171.09, 170.88, 168.87, 166.89, 91.15, 69.96, 66.82, 62.30, 52.58, 46.95, 20.90, 20.76. HRMS (ESI): m/z calculated for $C_{14}H_{24}N_5O_9$ $[M+NH_4]^+$ 406.1569, found 406.1569, m/z calculated for $C_{14}H_{20}N_4O_9Na$ $[M+Na]^+$ 411.1122, found 411.1128.

Synthesis of 4,6-isopropyidene-*N*-azidoacetylgalactosamine (9)

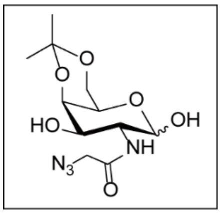

GalNAz (200 mg, 0.76 mmol) was added to 5 mL acetone at 0 °C in an ice-water bath, then 2,2-dimethoxypropane (0.94 mL, 763 mmol) and (±)-camphorsulfonic acid (17.7 mg, 7.63 mmol) were added. The reaction system was warmed to 4 °C and stirred vigorously until starting materials were dissolved (about 1 h). The reaction was quenched by triethylamine, followed by removal of solvent *in vacuo*. The residue was purified by flash chromatography, eluted with EtOAc:PE gradually based the volume ratio from 1:1 to 4:1 to give a white amorphous solid (R_f=0.2, EA). The product was further purified to remove 3,4-isopropyidene-*N*-azidoacetylgalactosamine by reverse-phase HPLC (XBridge Prep C18, 5 μm, OBD 30×25 mm column), eluting with acetonitrile from 5% to 100% (vol/vol) and water during 12 min at a flow rate of 25 mL/min, to give the product **9** as white solid (110 mg, 48%, α/β, 3:1). ^{1}H NMR (500 MHz, CD_3OD) δ 5.23 (d, J=3.5 Hz, 1H), 4.67 (d, J=8.5Hz, 1H), 4.33 (dd, J=11.0, 3.5Hz, 1H), 4.28 (d, J=4.0 Hz, 1H), 4.24-4.16 (m, 3H), 4.03-3.92 (m, 6H), 3.89-3.75 (m, 4H), 3.46 (s, 1H), 1.53 (s, 6H), 1.47 (s, 6H). ^{13}C NMR (125 MHz, CD_3OD) δ 169.69, 169.14, 98.74, 98.64, 95.23, 91.73, 70.08, 68.60, 68.00, 66.47, 66.40, 62.65, 62.54, 62.21, 54.32, 51.78, 51.52, 50.55, 28.28, 28.18, 17.60, 17.53. HRMS (ESI): m/z calculated for $C_{11}H_{19}N_4O_6$ $[M+H]^+$ 303.1299, found 303.1294.

Synthesis of 1,3-acetylated-4,6-isopropyidene-*N*-azidoacetylgalactosamine (10)

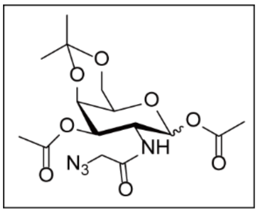

To a stirred solution of compound **9** (218 mg, 0.72 mmol) in 2 mL dehydrate pyridine, cooled to 0 °C in an ice-water bath, was added acetic anhydride (0.14 mL, 1.44 mmol). The reaction system was warmed to room temperature (R.T.) and stirred vigorously overnight, followed by removal of pyridine *in vacuo*. The residue was purified by flash chromatography with EA:PE gradually from the volume ratio at 1:4 to 1:1 to give the compound **10** (223 mg, 80%, α/β, 2.5:1) as white solid. ^{1}H NMR (500 MHz, $CDCl_3$) δ 6.33-6.24 (m, 3H), 5.78 (d, J=9.0 Hz, 1H), 5.20 (dd, J=11.5, 3.0 Hz, 1H), 5.11 (dd, J=11.5, 3.5 Hz, 1H), 4.83 (ddd, J=11.5, 9.5, 3.5 Hz, 1H), 4.44 (dt, J=11.5, 9.0 Hz, 1H), 4.32 (dd, J=3.3, 1.3 Hz 1H), 4.30 (dd, J=3.5, 1.0 Hz, 1H), 4.07-4.01 (m, 2H), 3.96-3.87 (m, 6H), 3.69 (q, J=1.7 Hz, 1H), 3.55 (q, J=1.7 Hz, 1H), 2.16-2.11 (m, 6H), 2.10 (m, 6H), 1.48-1.41 (m, 12H). ^{13}C NMR (125 MHz, $CDCl_3$) δ 171.74, 171.02, 169.71, 169.05, 166.97, 166.75, 99.12, 99.02, 92.37, 91.81, 70.88, 68.39, 67.11, 66.27, 65.88, 64.41, 62.39, 52.73, 52.61, 49.73, 46.70, 29.13, 20.98, 20.86, 18.68, 18.65. HRMS (ESI): m/z calculated for $C_{15}H_{26}N_5O_8$ $[M+NH_4]^+$ 404.1776, found 404.1777, m/z calculated for $C_{15}H_{22}N_4NaO_8$ $[M+Na]^+$ 409.1330, found 409.1337.

Synthesis of 1,3-acetylated-*N*-azidoacetylgalactosamine (3)

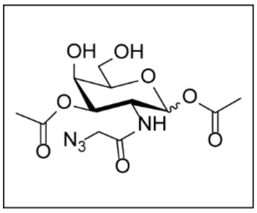

To a solution of compound **10** (135 mg, 0.35 mmol) dissolved in the mixture of acetonitrile and water (CH_3CN:H_2O=4:1, vol/vol) was added trifluoroacetic acid (52 μL, 0.7 mmol). The reaction mixture was stirred at R.T. for about 1 h, monitored by TLC until most of the starting material disappeared because too long reaction time would give acetyl group migrated products. The residue was evaporated under reduced pressure, purified by flash chromatography, and eluted with EtOAc:PE gradually from the volume ratio at 1:9 to 2:3 to produce the compound **3** (105 mg, 70%, α/β, 3.4:1) as white solid. ^{1}H NMR (500 MHz, $CDCl_3$) δ 6.97 (d, J=9.5 Hz, 1H), 6.55 (d, J=9.0 Hz, 1H), 6.19 (d, J=3.5 Hz, 1H), 5.76 (d, J=8.5 Hz, 1H), 5.17 (dd, J=11.3, 2.8 Hz, 1H), 5.09 (dd, J=11.0, 3.0 Hz, 1H), 4.79 (ddd, J=11.3, 9.0, 3.8 Hz, 1H), 4.52 (dt, J=11.0, 9.0 Hz, 1H), 4.22 (d, J=2.5 Hz, 1H), 4.15 (d, J=3.0 Hz, 1H), 3.95-3.52 (m, 14H), 2.16 (s, 3H), 2.12 (s, 3H), 2.09 (s, 3H), 2.08 (s, 3H). ^{13}C NMR (125 MHz, $CDCl_3$) δ 171.60, 171.06,

170.00, 169.75, 167.97, 167.67, 92.96, 91.30, 75.20, 72.80, 72.18, 70.26, 67.96, 66.88, 62.32, 61.71, 52.55, 52.43, 49.61, 47.07, 20.99, 20.93, 20.90, 20.83. HRMS (ESI): m/z calculated for $C_{12}H_{19}N_4O_8$ $[M+H]^+$ 347.1197, found 347.1194.

Synthesis of 1,3-propionyl-4,6-isopropyidene-*N*-azidoacetylgalactosamine (11)

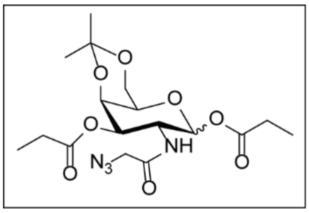

To a solution of compound **9** (133 mg, 0.44 mmol) dissolved in 2 mL dry pyridine was added propionic anhydride (0.23 mL, 1.79 mmol) at 0 °C. The reaction mixture was warmed to R.T. and stirred overnight. After removal of pyridine *in vacuo*, the residue was purified by flash chromatography with EtOAc:PE gradually from the volume ratio 1:4 to 1:1 to provide the white solid compound **11** (149 mg, 82%, α/β, 3.6:1). ^{1}H NMR (500 MHz, $CDCl_3$) δ 6.31 (d, J=3.5 Hz, 1H), 6.28-6.20 (m, 2H), 5.78 (d, J=8.5 Hz, 1H), 5.20 (dd, J=11.3, 3.3 Hz, 1H), 5.10 (dd, J=11.3, 3.3 Hz, 1H), 4.85 (ddd, J=11.5, 9.0, 3.5 Hz, 1H), 4.47 (dt, J=11.0, 9.0 Hz, 1H), 4.33 (dd, J=3.3, 1.3 Hz, 1H), 4.30 (dd, J=3.5, 1.0 Hz, 1H), 4.07-4.00 (m, 2H), 3.96-3.87 (m, 6H), 3.68 (q, J=1.7 Hz, 1H), 3.55 (q, J=1.6 Hz, 1H), 2.45-2.34 (m, 8H), 1.48 (s, 6H), 1.42 (m, 6H), 1.21-1.10 (m, 12H). ^{13}C NMR (125 MHz, $CDCl_3$) δ 175.28, 174.58, 173.25, 172.51, 166.81, 166.60, 99.07, 98.97, 92.37, 91.70, 70.76, 68.29, 67.15, 66.23, 65.83, 64.40, 62.43, 62.39, 52.73, 52.63, 49.69, 46.70, 29.15, 29.11, 27.65, 27.57, 27.49, 18.64, 9.23, 8.96, 8.74. HRMS (ESI): m/z calculated for $C_{17}H_{27}N_4O_8$ $[M+H]^+$ 415.1823, found 415.1822.

Synthesis of 1,3-propionyl-*N*-azidoacetylgalactosamine (4)

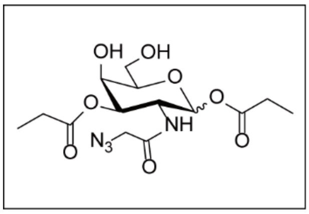

Compound **11** (177 mg, 0.43 mmol) was dissolved in the mixture of acetonitrile and water (CH_3CN:H_2O = 4:1, vol/vol). Trifluoroacetic acid (64 μL, 0.86 mmol) was added dropwise, followed by stirring at R.T. 1h. Reaction was concentrated and purified by flash chromatography, using EtOAc (10%-40%, vol/vol) and PE as eluent, to give compound **4** (145 mg, 74%, α/β, 5:1) as white solid. ^{1}H NMR (500 MHz, $CDCl_3$) δ 6.80 (d, J=9.5 Hz, 1H), 6.45 (d, J=9.0 Hz, 1H), 6.22 (s, 1H), 5.77 (d, J=9.0 Hz, 1H), 5.19 (dd, J=11.3, 2.8 Hz, 1H), 5.13-5.09 (m, 1H), 4.85-4.77 (m, 1H), 4.61-4.53 (m, 1H), 4.21 (s, 1H), 4.15 (s, 1H), 4.08-3.75 (m, 12H), 3.39 (br, 2H), 2.46-2.34 (m, 8H), 1.20-1.08 (m, 12H). ^{13}C NMR (125 MHz, $CDCl_3$) δ 174.99, 174.36,

173.27, 172.92, 167.57, 167.26, 92.87, 91.12, 77.25, 75.08, 72.52, 72.05, 70.05, 68.17, 67.09, 62.46, 61.84, 52.52, 52.43, 49.62, 47.08, 27.53, 27.45, 27.40, 27.36, 9.01, 8.99, 8.89, 8.66. HRMS (ESI): m/z calculated for $C_{14}H_{23}N_4O_8$ $[M+H]^+$ 375.1510, found 375.1508, m/z calculated for $C_{14}H_{26}N_5O_8$ $[M+NH_4]^+$ 392.1776, found 392.1773.
