# Supplementary material for: Next-generation unnatural monosaccharides reveal that ESRRB O-GlcNAcylation regulates pluripotency of mouse embryonic stem cells
Source: Nat Commun. 2019 Sep 6;10:4065. doi: 10.1038/s41467-019-11942-y (PMC6731260; doi:10.1038/s41467-019-11942-y)
Supplement: Supplementary file 3 — Description of Additional Supplementary Files [file 41467_2019_11942_MOESM3_ESM.pdf]

Description of Additional Supplementary Files

File Name: Supplementary Data 1

Description: Raw data of O-GlcNAz sites identified with 1,3-Ac_2GalNAz in HeLa cells.

File Name: Supplementary Data 2

Description: Raw data of S-HexNAz sites identified with 1,3-Ac_2GalNAz in HeLa cells.

File Name: Supplementary Data 3

Description: Raw data of S-AcHexNAz sites identified with 1,3-Ac2GalNAz in HeLa cells.

File Name: Supplementary Data 4

Description: Raw data of O-GlcNAz sites identified with 1,3-Pr_2GalNAz in HeLa cells.

File Name: Supplementary Data 5

Description: Raw data of S-HexNAz sites identified with 1,3-Pr_2GalNAz in HeLa cells.

File Name: Supplementary Data 6

Description: Raw data of S-PrHexNAz sites identified with 1,3-Pr_2GalNAz in HeLa cells.

File Name: Supplementary Data 7

Description: Raw data of gene expression analysis in mESCs stably expressing WT and S25A ESRRB by RNA sequencing.
