# Supplementary material for: Next-generation unnatural monosaccharides reveal that ESRRB O-GlcNAcylation regulates pluripotency of mouse embryonic stem cells
Source: Nat Commun. 2019 Sep 6;10:4065. doi: 10.1038/s41467-019-11942-y (PMC6731260; doi:10.1038/s41467-019-11942-y)
Supplement: Supplementary file 11 — Reporting Summary [file 41467_2019_11942_MOESM11_ESM.pdf]

nature research

Corresponding author(s): Xing Chen

Last updated by author(s): Aug 1, 2019

Reporting Summary

Nature Research wishes to improve the reproducibility of the work that we publish. This form provides structure for consistency and transparency in reporting. For further information on Nature Research policies, see Authors & Referees and the Editorial Policy Checklist.

Statistics

For all statistical analyses, confirm that the following items are present in the figure legend, table legend, main text, or Methods section.

n/a	Confirmed	
☐	☒	The exact sample size (*n*) for each experimental group/condition, given as a discrete number and unit of measurement
☐	☒	A statement on whether measurements were taken from distinct samples or whether the same sample was measured repeatedly
☐	☒	The statistical test(s) used AND whether they are one- or two-sided<br>*Only common tests should be described solely by name; describe more complex techniques in the Methods section.*
☒	☐	A description of all covariates tested
☒	☐	A description of any assumptions or corrections, such as tests of normality and adjustment for multiple comparisons
☐	☒	A full description of the statistical parameters including central tendency (e.g. means) or other basic estimates (e.g. regression coefficient) AND variation (e.g. standard deviation) or associated estimates of uncertainty (e.g. confidence intervals)
☐	☒	For null hypothesis testing, the test statistic (e.g. *F*, *t*, *r*) with confidence intervals, effect sizes, degrees of freedom and *P* value noted<br>*Give P values as exact values whenever suitable.*
☒	☐	For Bayesian analysis, information on the choice of priors and Markov chain Monte Carlo settings
☒	☐	For hierarchical and complex designs, identification of the appropriate level for tests and full reporting of outcomes
☒	☐	Estimates of effect sizes (e.g. Cohen's *d*, Pearson's *r*), indicating how they were calculated

Our web collection on statistics for biologists contains articles on many of the points above.

Software and code

Policy information about availability of computer code

Data collection	The proteome MS data were obtained from Thermo Xcalibur 2.2 SP1.48 and the NMR data were acquired using TopSpin 3.2. FLIM-FRET imaging was performed on a TCS SP8X scanning confocal microscope (Leica). Fluorescent images of supplementary fig. 13 were collected using a DMI4000 B inverted microscope (Leica). In-gel fluorescence scanning was conducted from a Typhoon FLA 9500 (GE Healthcare). Images of Coomassie Brilliant Blue-stained gels were collected on a ChemiDoc XRS+ (Bio-Rad). Western blotting and streptavidin blotting were acquired from a Tanon-5200Multi (Tanon). The quantitative RT-PCR data were obtained from a ViiA 7 (Applied Biosystems). LC-MS detection was performed using Waters ACQUITY UPLC I-Class SQD 2 MS spectrometer with electrospray ionization (ESI). RNA sequencing was performed on Illumina Hiseq X Ten.
Data analysis	GraphPad Prism 6.0, Origin 8.0 and Microsoft Excel for statistics. Image J (version 1.52a) and Photoshop CC 2018 for image analysis. MaxQuant (version 1.5.8.2) for database searches. TCSPC software (SymPhoTime 64 software, PicoQuant GmbH) for FLIM-FRET image analysis. SAMtools v1.3, Picard tools v1.140, featureCounts v1.6.3 and DESeq2 v1.20.0 for RNA-seq data analysis.

For manuscripts utilizing custom algorithms or software that are central to the research but not yet described in published literature, software must be made available to editors/reviewers. We strongly encourage code deposition in a community repository (e.g. GitHub). See the Nature Research guidelines for submitting code & software for further information.

Data

Policy information about availability of data

All manuscripts must include a data availability statement. This statement should provide the following information, where applicable:

- Accession codes, unique identifiers, or web links for publicly available datasets
- A list of figures that have associated raw data
- A description of any restrictions on data availability

All data generated or analyzed during this study are available from the corresponding authors on reasonable request.

Field-specific reporting

Please select the one below that is the best fit for your research. If you are not sure, read the appropriate sections before making your selection.

☒ Life sciences ☐ Behavioural & social sciences ☐ Ecological, evolutionary & environmental sciences

For a reference copy of the document with all sections, see nature.com/documents/nr-reporting-summary-flat.pdf

Life sciences study design

All studies must disclose on these points even when the disclosure is negative.

Sample size	At least three independent replicates were performed. For the teratoma formation assay, ten independent experiments were performed.
Data exclusions	No data were excluded from the analyses.
Replication	Yes, three biological replicates at least.
Randomization	Independent experiments such as immunoblotting and in-gel fluorescence scanning cannot be randomized. For the teratoma formation assay and HE staining assay, mice of 8-week were randomly allocated into different groups.
Blinding	For imaging related assays, data were unbiasedly collected and analysed by De-en Sun and Yi Hao. For the teratoma formation assay, Xinqi Fan and Yi Hao, who collected the data were blind to the genotypes and treatments.

Reporting for specific materials, systems and methods

We require information from authors about some types of materials, experimental systems and methods used in many studies. Here, indicate whether each material, system or method listed is relevant to your study. If you are not sure if a list item applies to your research, read the appropriate section before selecting a response.

Materials & experimental systems

n/a	Involved in the study	
☐	☒	Antibodies
☐	☒	Eukaryotic cell lines
☒	☐	Palaeontology
☐	☒	Animals and other organisms
☒	☐	Human research participants
☒	☐	Clinical data

Methods

n/a	Involved in the study	
☒	☐	ChIP-seq
☒	☐	Flow cytometry
☒	☐	MRI-based neuroimaging

Antibodies

Antibodies used	Antibodies included anti-ESRRB (Abclonal, A13977, 1:500), anti-DDDDK-tag (MBL, M185-3, 1:5,000), anti-GFP (Abcam, ab183734, 1:5,000), anti-HIS (CST, 12698, 1:3,000), anti-RL2 (Abcam, ab2739, 1:1,000), anti-OGT (Abcam, ab177941, 1:2,000), anti-HA (CST, 3724, 1:3,000), anti-GAPDH-HRP (Sigma-Aldrich, G9295, 1:10,000), anti-streptavidin-HRP (Beyotime, A0303, 1:3,000), anti-mouse IgG-HRP (Abcam, ab97023, 1:5,000), anti-rabbit IgG-HRP (Abcam, ab6721, 1:5,000).
Validation	All antibodies are validated by vendors indicated above.

Eukaryotic cell lines

Policy information about cell lines

Cell line source(s)	R1 murine embryonic stem cells were kindly provided by Prof. Ye-Guang Chen at Tsinghua University. HEK 293FT cells were kindly provided by Prof. Qin Shen at Tongji University. HeLa (ATCC® CCL-2), CHO (ATCC® CCL-61), Neuro-2a (ATCC® CCL-131), HT-1080 (ATCC® CCL-121), SH-SY5Y (ATCC® CRL-2266), NCI-H1299 (ATCC® CRL-5803), A549 (ATCC® CCL-185), NIH/3T3 (ATCC® CRL-1658), MCF-7 (ATCC® HTB-22), HEK293T (ATCC® CRL-11268) cells were obtained from American Type Culture Collection.
Authentication	No further authentication was performed.
Mycoplasma contamination	All cell lines were tested negative for mycoplasma contamination.
Commonly misidentified lines (See ICLAC register)	None.

Animals and other organisms

Policy information about studies involving animals; ARRIVE guidelines recommended for reporting animal research

Laboratory animals	8-week old male BALB/c nude mice were from Charles River Laboratories and kept in a specific-pathogen-free facility at Beijing Laboratory Animal Research Center (BLARC). Mouse-related experiments were conducted in accordance with institutional guidelines.
Wild animals	The study did not involve wild animals.
Field-collected samples	The study did not involve samples collected from field.
Ethics oversight	Beijing Laboratory Animal Research Center (BLARC).

Note that full information on the approval of the study protocol must also be provided in the manuscript.
